# Synaptogenic effect of *APP*-Swedish mutation in familial Alzheimer’s disease

**DOI:** 10.1126/scitranslmed.abn9380

**Published:** 2022-10-19

**Authors:** Bo Zhou, Jacqueline G. Lu, Alberto Siddu, Marius Wernig, Thomas C. Südhof

**Affiliations:** 1 Department of Molecular and Cellular Physiology, Stanford University School of Medicine, 265 Campus Drive, Stanford, CA 94305, USA.; 2 Department of Pathology, Stanford University School of Medicine, 265 Campus Drive, Stanford, CA 94305, USA.; 3 Institute for Stem Cell Biology and Regenerative Medicine, Stanford University School of Medicine, 265 Campus Drive, Stanford, CA 94305, USA.; 4 Howard Hughes Medical Institute, Stanford University School of Medicine; Stanford 94305, USA

## Abstract

Mutations in β-amyloid (Aβ) precursor protein (*APP*) cause familial Alzheimer’s disease (AD) probably by enhancing Aβ peptides production from APP. An antibody targeting Aβ (aducanumab) was approved as an AD treatment; however, some Aβ antibodies have been reported to accelerate, instead of ameliorating, cognitive decline in individuals with AD. Using conditional *APP* mutations in human neurons for perfect isogenic controls and translational relevance, we found that the *APP*-Swedish mutation in familial AD increased synapse numbers and synaptic transmission, whereas the *APP* deletion decreased synapse numbers and synaptic transmission. Inhibition of BACE1, the protease that initiates Aβ production from APP, lowered synapse numbers, suppressed synaptic transmission in wild-type neurons, and occluded the phenotype of *APP*-Swedish–mutant neurons. Modest elevations of Aβ, conversely, elevated synapse numbers and synaptic transmission. Thus, the familial AD-linked *APP*-Swedish mutation under physiologically relevant conditions increased synaptic connectivity in human neurons via a modestly enhanced production of Aβ. These data are consistent with the relative inefficacy of BACE1 and anti-Aβ treatments in AD and the chronic nature of AD pathogenesis, suggesting that AD pathogenesis is not simply caused by overproduction of toxic Aβ but rather by a long-term effect of elevated Aβ concentrations.

## INTRODUCTION

Alzheimer’s disease (AD) is the major cause of dementia worldwide, affecting millions of people. A key feature of AD is the formation of plaques containing β-amyloid (Aβ) peptides (1–4). Aβ is produced by site-specific cleavage of amyloid precursor protein (APP) mediated by two proteases, β-secretase 1 (BACE1) and γ-secretase (5–6). Mutations in APP and in γ-secretase alter production of Aβ and cause familial AD, suggesting a role for Aβ in AD pathogenesis (4). These findings prompted the development of antibodies to Aβ as a therapeutic approach, culminating in the recent approval by the U.S. Food and Drug Administration of an Aβ antibody (aducanumab) as a treatment for AD. However, the approval of aducanumab raised controversies due to its unclear therapeutic efficacy (7), especially because an antibody to soluble Aβ worsened the cognitive decline in individuals with AD in other clinical trials (8). Similarly, verubecestat and atabecestat, highly potent small-molecule inhibitors of BACE1, failed to ameliorate AD progression but caused a faster clinical decline (5, 9–10). These clinical data suggest that APP cleavage and Aβ production may have important physiological functions. However, these functions are poorly understood; even the physiological role of the precursor protein APP is largely unknown. The lack of insight into the underlying biology of key genes such as APP has hindered progress in understanding AD (11). For example, to this date, it is uncertain whether presenilin mutations causing familial AD are pathogenic because the mutated catalytic subunits of γ-secretase increase the production of Aβ or change the type of generated Aβ species or because presenilin mutations induce a partial loss of function of γ-secretase (12).

APP, Aβ, and presenilins have been extensively studied in mouse models, providing convincing evidence that high Aβ concentrations are toxic to synapses. For example, transgenic mice overexpressing human APP carrying the so called “Swedish” mutation (K595N/M596L) that increases Aβ and causes early-onset AD in patients (13) exhibit synapse loss and a decrease in synaptic transmission (14–16). Moreover, addition of Aβ to murine cultured neurons or brain slices is toxic to synapses (17–21). Several studies showed that exogenous Aβ promotes glutamate receptor endocytosis and blocks long-term potentiation (LTP) and memory formation (22–27). Other studies, however, reported positive roles of Aβ at synapses. For example, Aβ increases Ca^2+^ influx in cultured neurons and activates synaptic transmission and presynaptic facilitation in mouse hippocampal cultures and slices (28–31), and Aβ is required in young rats for memory stabilization (32). Moreover, studies of neurons generated from patient-derived induced pluripotent stem (iPS) cells with an *APP*-Swedish mutation revealed increases in the amplitudes and frequency of spontaneous miniature excitatory postsynaptic potentials (mEPSCs) (33). Furthermore, deletion of *APP* in mice may decrease dendritic spine numbers and reduce LTP (34). The diverse outcomes of these studies on Aβ could be due to the use of different concentrations, forms, and sources of Aβ. Alternatively, rodents may not be an appropriate model for studying the role of Aβ in human health, given the exceptional vulnerability of humans to AD (35) and relatively poor evolutionary conservation of the Aβ sequence. Because of these constraints, extensive studies on human iPS cells and neurons were performed, with hundreds of papers published at present (36–37). However, most of these papers focus on creating AD disease models, and few examined the biological roles and pathological dysfunction of APP or Aβ. Human neurons carrying familial AD-associated *APP* mutations exhibited elevated Aβ and a modest increase in the size of Rab5-positive endosomes (38), but other features of these neurons, such as their overall development or synaptic connections, were not examined in depth. To address these gaps in our knowledge, we here examined the role of APP and Aβ in human neurons using a tightly controlled genetic approach coupled with a functional electrophysiological analysis.

## RESULTS

### Generation of human neurons with a conditional Swedish mutation in the *APP* gene

**Fig. 1.**
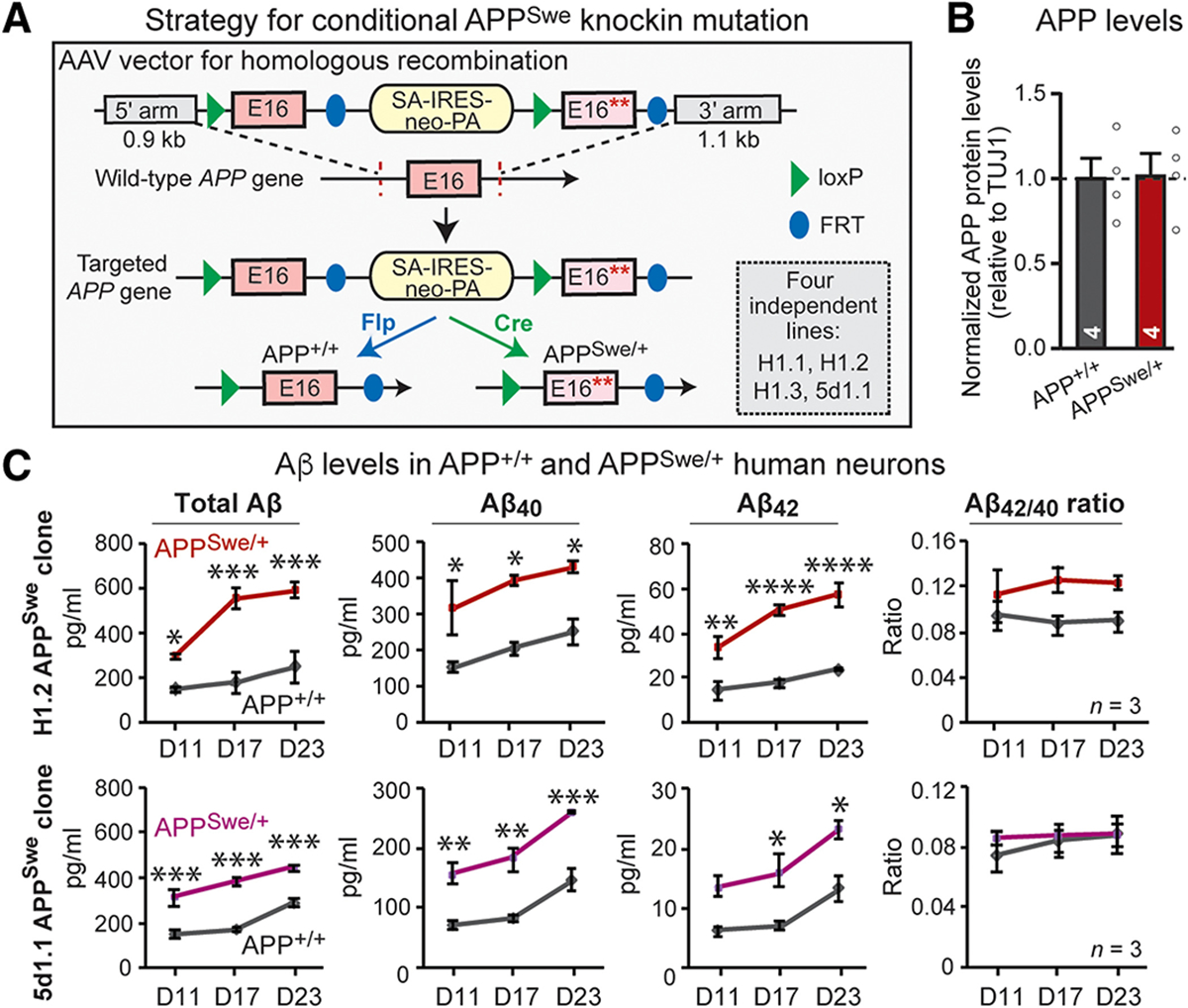
Generation of conditional Swedish-mutant *APP*^Swe/+^ human neurons with APP expression and Aβ secretion measurements. (A) Gene-targeting strategy. The mutant allele, inserted by homologous recombination into hES and iPS cells, contains a duplicated exon 16 (E16), with one of the copies including the Swedish mutation (asterisks = Swedish point mutation). The duplicated exons are flanked by FRT and loxP sites, such that Flp and Cre recombination induces retention of either only the wild-type (*APP*^+/+^) or the Swedish-mutant exon (*APP*^Swe/+^), respectively. See fig. S1 for details. (B) Immunoblotting analysis of APP expression on wild-type *APP*^+/+^ and Swedish-mutant *APP*^Swe/+^ human neurons derived from H1.2 stem cells. For representative blots, see fig. S3E. (C) Assessment of secreted Aβ peptides by enzyme-linked immunosorbent assay (ELISA) on supernatants harvested from *APP*^+/+^ and *APP*^Swe/+^ neuron cultures at day 11 (D11), day 17 (D17), or day 23 (D23) after induction of independent stem cell clones (top, H1.2; bottom, 5d1.1). All data are from human neurons analyzed 5 weeks after neuronal induction unless noted. Analyses of additional clones, representative immunoblots, and further images are shown in fig. S3. All numerical data are means ± SEM; numbers of biological replicates are indicated in the graphs. Statistical significance was assessed by two-way ANOVA with post hoc corrections (C) or Student’s t test (B), with *P < 0.05, **P < 0.01, ***P < 0.001, and ****P < 0.0001. Nonsignificant comparisons are not indicated.

Among *APP* mutations associated with familial AD, the Swedish mutation (K595N/M596L; located in exon 16 of *APP*) has been studied most intensely because it promotes β-secretase over α-secretase cleavage of APP and thereby increasing Aβ production (13, 39–40). Although many studies were performed on neurons derived from patients’ iPS cells with familial AD (41–44), such experiments are difficult to perform under truly isogenic conditions. Introduction or reversal of disease mutations into iPS cells by genetic engineering produces genetically similar cell lines (38, 44), but these procedures require clonal selection and passaging of cells that induce genetic changes. Therefore, to investigate the effect of the *APP*-Swedish mutation under truly isogenic conditions, we introduced the Swedish mutation conditionally in human embryonic stem (hES) and iPS cells, thereby avoiding clonal variation (Fig. 1A). Using homologous recombination, we inserted into the *APP* gene tandem copies of exon 16 that were either wild type (WT) or carried the Swedish mutation flanked by loxP and FRT sites (Fig. 1A and fig. S1; see Materials and Methods for details). As a result, Flp recombination excises the mutant but not WT exon 16, whereas Cre recombination excises the WT but not mutant exon 16; without Flp or Cre recombination, the engineered *APP* allele represents a loss-of-function state (Fig. 1A). Using this strategy, we generated four correctly targeted hES and iPS cell lines. We then derived excitatory neurons from these clones using forced expression of neurogenin 2 (Ngn2) (see fig. S2 for characterization) (45) and infected the developing postmitotic neurons with viruses expressing Flp or Cre recombinase. Quantifications confirmed a nearly complete infection rate (fig. S3, B and C). In this manner, we produced human neurons that are either homozygous for the WT *APP* gene or heterozygous for the Swedish-mutant and the WT *APP* gene but contain an otherwise identical genetic background.

**Fig. 2.**
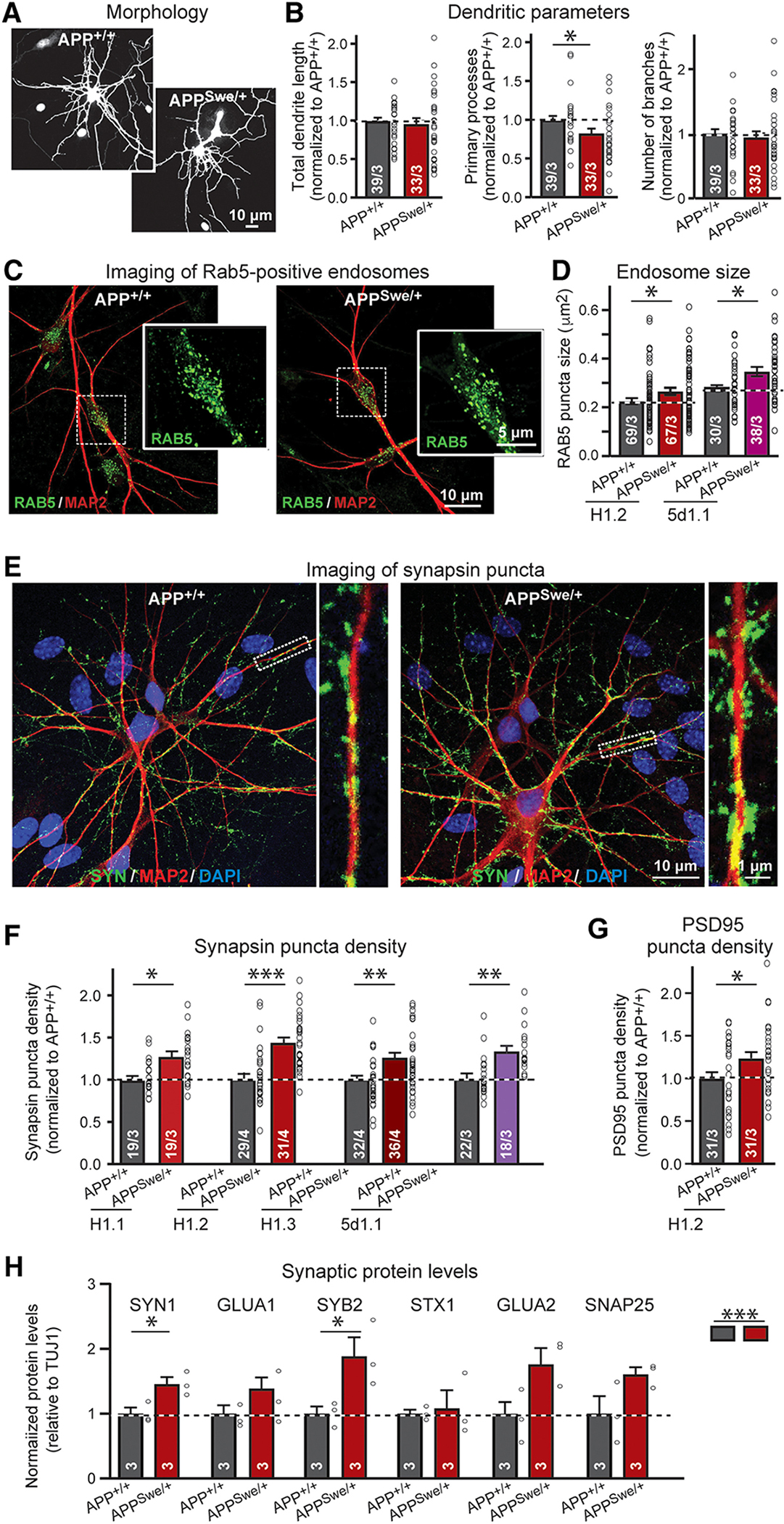
Characterization of dendrite development, endosome sizes, and synapse densities in human conditional Swedish-mutant *APP*^Swe/+^ neurons compared to their perfect isogenic controls. (A and B) Dendritic arborization of wild-type *APP*^+/+^ and Swedish-mutant *APP*^Swe/+^ human neurons [(A) representative images of human neurons sparsely transfected with GFP; (B) quantification of selected dendritic parameters of neurons derived from H1.2 stem cells). (C and D) Rab5-positive endosome sizes in wild-type *APP*^+/+^ and Swedish-mutant *APP*^Swe/+^ human neurons [(C) representative images; insets show higher magnification views of endosomes; (D) summary graphs of endosome sizes in neurons obtained from two independent stem cell clones (left, H1.2; right, 5d1.1)]. (E to G) Quantification of synapsin- and PSD95-positive synaptic puncta in isogenic *APP*^+/+^ and *APP*^Swe/+^ human neurons [(E) representative images of wild-type *APP*^+/+^ and Swedish-mutant *APP*^Swe/+^ human neurons immunolabeled for the presynaptic marker synapsin (SYN; green) and the dendritic marker MAP2 (red); left: overviews; right: higher-magnification images (from clone H1.2); (F) summary graphs of the synapsin-puncta density determined in neurons derived from four independent conditionally Swedish-mutant stem cell clones; (G) summary graph of the synapse density measured by PSD95 immunocytochemistry in wild-type and Swedish-mutant human neurons derived from clone H1.2]. For further representative images, see fig. S4. DAPI, 4′,6-diamidino-2-phenylindole. (H) Immunoblotting analysis of the synaptic proteins expressed in human wild-type *APP*^+/+^ and Swedish-mutant *APP*^Swe/+^ neurons (for representative immunoblots, see fig. S4D). All neurons were analyzed 5 weeks after neuronal induction; additional data are shown in figs. S3 and S4. All numerical data are means ± SEM; numbers of images/experiments (B, D, F, and G) or of experiments (H) are indicated in the bars. Statistical significance was assessed by two-way ANOVA with post hoc corrections (H) or Student’s t test (all other bar graphs), with*P < 0.05, **P < 0.01, and ***P < 0.001. Nonsignificant comparisons are not indicated. GLUA1, glutamate receptor 1; SYB2, synaptobrevin 2; STX1, syntaxin 1; GLUA2, glutamate receptor 2; SNAP25, synaptosomal-associated protein 25.

Swedish-mutant human neurons exhibited no survival phenotype (fig. S3, A to C) and expressed the same amount of APP as WT neurons (Fig. 1B and fig. S3, D and E) but produced more Aβ_40_ and Aβ_42_ than WT neurons (Fig. 1C). Swedish-mutant neurons exhibited a small increase in phosphorylated Tau protein in human neurons (fig. S3, F to I) but no major changes in dendritic arborization or soma size (Fig. 2, A and B, and fig. S3, J to L). Imaging of Rab5-positive endosomes, however, detected a modest but consistent increase (15 to 20%) in the size of Rab5-positive endosomes in both hES and iPS cell–derived neurons (Fig. 2, C and D, and fig. S3M), confirming previous observations with *APP*-Swedish–mutant human neurons generated with a different protocol (38).

### The Swedish mutation of *APP* increases functional synapse numbers

We next imaged synapses in WT and Swedish-mutant human neurons by immunocytochemistry for synapsin (Fig. 2E and fig. S4A). We observed an increased (~20%) synapse density in human neurons carrying the Swedish mutation. This increase was detected in neurons derived from all conditionally mutant hES and iPS cells (Fig. 2F and fig. S4B) and was confirmed by staining neurons for PSD95 as a postsynaptic marker (Fig. 2G and fig. S4C). Moreover, analyses of the protein composition of Swedish-mutant human neurons revealed a relatively large elevation (20 to 80% depending on the protein) in the expression of synaptic marker proteins (Fig. 2H and fig. S4D), consistent with an increase in synapse numbers.

**Fig. 3.**
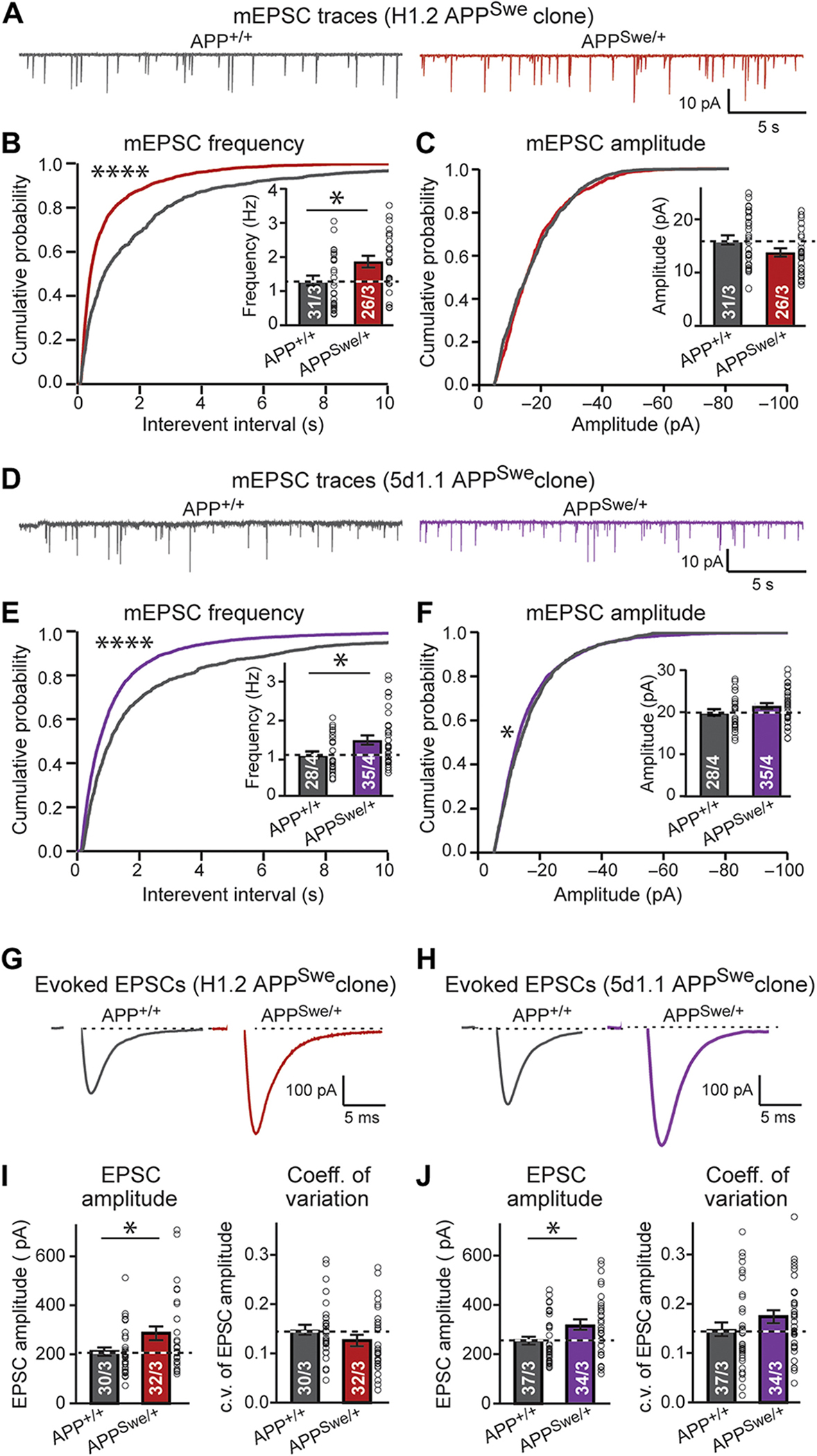
Synaptic transmission in human wild-type *APP*^+/+^ and Swedish-mutant *APP*^Swe/+^ neurons. (A to F) Assessment of the frequency and amplitude of spontaneous miniature excitatory postsynaptic currents (mEPSCs), monitored in the presence of tetrodotoxin in *APP*^+/+^ and *APP*^Swe/+^ human neurons derived from H1.2 (A to C) and 5d1.1 clones (D to F) [(A and D) representative traces; (B and E) cumulative probability plots of the interevent times and summary graph of the mEPSC frequency (inset); (C and F) cumulative probability plots and summary graphs (inset) of mEPSC amplitudes]. (G to J) Assessment of the amplitude and coefficient of variation of the evoked EPSCs measured on isogenic *APP*^+/+^ and *APP*^Swe/+^ human neurons derived from H1.2 (G and I) and 5d1.1 clones (H and J) [(G and H) representative traces; (I and J) summary graphs of EPSC amplitudes (left) and coefficients of variation (c.v.) (right)]. All neurons were analyzed 5 weeks after neuronal induction; additional data are shown in fig. S4. All numerical data are means ± SEM; numbers of cells/experiments analyzed are indicated in bars. Statistical significance was assessed by Kolmogorov-Smirnov (KS) test (all cumulative probability plots) or Student’s t test (all bar graphs), with*P < 0.05 and ****P < 0.0001. Nonsignificant comparisons are not indicated.

The increase in synapses induced by the *APP*-Swedish mutation is unexpected because the Swedish mutation leads to a two- to three-fold elevation in Aβ production (Fig. 1C), which is thought to be neuro- and synaptotoxic. Thus, we expected a decrease instead of an increase in synapses (46–48). Although the increase in synapses was found using two independent approaches (imaging and protein expression measurements), we sought to further validate this result using electrophysiological recordings. Measurements of the frequency and amplitude of spontaneous mEPSCs in isogenic WT and Swedish-mutant neurons derived from independent stem cell clones demonstrated a robust increase (~30%) in mEPSC frequency without a change in mEPSC amplitude, consistent with the observed elevation of synapse numbers (Fig. 3, A to F). In addition, recordings of EPSCs evoked by action potentials documented a similar increase in synaptic strength, monitored as the EPSC amplitude, without a significant change in the coefficient of variation as a measure of the release probability (p > 0.05; Fig. 3, G to J). Viewed together, these data establish with multiple independent approaches that under isogenic conditions, the Swedish mutation in *APP* induces an increase in the number of functional synapses in human neurons.

### BACE1 inhibition blocks the effect of the Swedish mutation of *APP* in human neurons

The key consequence of the Swedish mutation is thought to be an enhancement of APP cleavage by BACE1, leading to increased Aβ production (39–40, 49), as also observed in our experiments using isogenic human neurons (Fig. 1C). The unexpected effect of the Swedish mutation on synapse numbers suggests that, contrary to its supposedly synaptotoxic effect, Aβ may support synapses. If correct, then this hypothesis would imply a central role for BACE1 in regulating synapses. To test this hypothesis, we asked whether pharmacological inhibition of BACE1 alters synaptic function and whether a potential effect of BACE1 inhibition is altered by the Swedish mutation in *APP*.

**Fig. 4.**
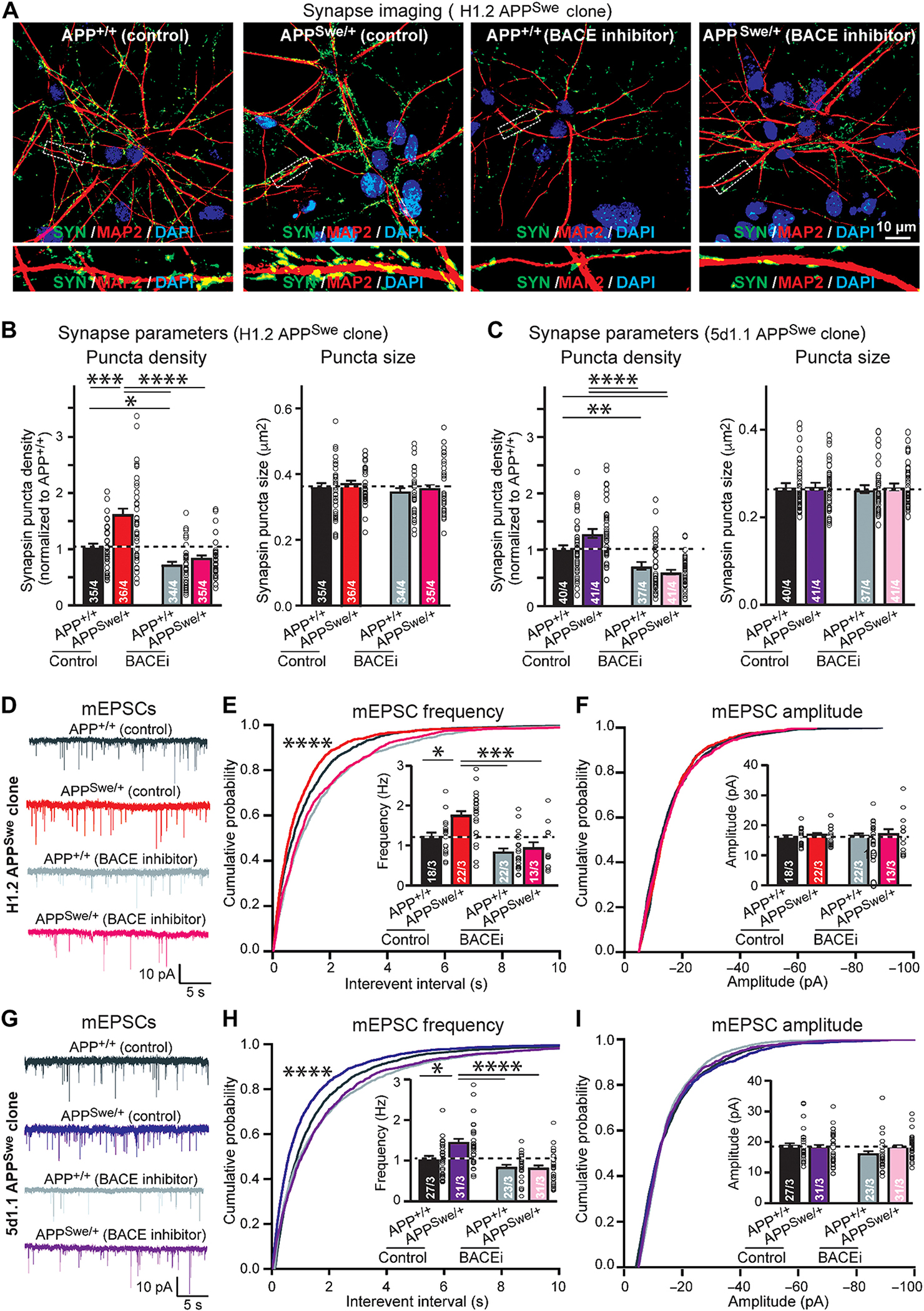
The effect of pharmacological BACE1 inhibition on synapse formation and synaptic transmission in human wild-type *APP*^+/+^ and Swedish-mutant *APP*^Swe/+^ neurons. (A to C) Quantification of synapse density in WT *APP*^+/+^ and Swedish-mutant *APP*^Swe/+^ neurons with or without BACE1 inhibition measured by immunocytochemistry for synapsin in human neurons derived from two independent stem cell clones with conditional Swedish mutations [(A) representative images of *APP*^+/+^ and *APP*^Swe/+^ human neurons with or without BACE1 inhibitor (BACEi) treatment; neurons were stained for synapsin (SYN; green) and MAP2 (red; bottom: high magnification of dendrites); (B and C) summary graphs of the density (left) and size (right) of synapsin-positive synaptic puncta in neurons derived from H1.2 (B) or 5d1.1 cells (C)]. Neurons were treated with the BACE1 inhibitor LY2886721 at 2 μM starting 1 week after neuronal induction and analyzed 4 weeks later. (D to I), The frequency and amplitude of mEPSCs in isogenic *APP*^+/+^ and *APP*^Swe/+^ neurons with or without BACE1 inhibition monitored by whole-cell patch-clamp recordings in human neurons derived from two independent stem cell clones [H1.2 (D to F) and 5d1.1 (G to I)] with conditional *APP*-Swedish mutations [(D and G) representative mEPSC traces; (E and H) cumulative probability plots of the mEPSC interevent interval and summary graph of the mEPSC frequency (inset); (F and I) cumulative probability plot and summary graph (inset) of the mEPSC amplitude]. All numerical data are means ± SEM (cells/experiments are indicated in all bars); statistical significance was assessed by two-way ANOVA with post hoc corrections (all bar graphs) or KS test (all cumulative probability plots), with*P < 0.05, **P < 0.01, ***P < 0.001, and ****P < 0.0001. Nonsignificant comparisons are not indicated.

Chronic treatment of WT human neurons with the BACE1 inhibitor LY2886721 (50–51) suppressed production of Aβ and of the secreted extracellular domains of APP that contain a BACE1-generated C-terminal sequence (sAPPβ) (fig. S5, A to C). Chronic BACE1 inhibition did not lower the secreted extracellular domains of APP that contain an α-secretase–generated C-terminal sequence (sAPPα and had no effect on APP expression, demonstrating its selectivity (fig. S5, C and D). Chronic BACE1 inhibition robustly decreased synapse numbers (~30%) in WT neurons, as measured by synapsin immunocytochemistry, without affecting synapse size (Fig. 4, A to C). Moreover, chronic BACE1 inhibition completely blocked the increase in synapse numbers induced by the conditional heterozygous *APP*-Swedish mutation, again without affecting the synapse size (Fig. 4, A to C).

To independently confirm these results suggesting that the synaptic effects of the Swedish mutation in *APP* require BACE1 cleavage, we measured spontaneous synaptic activity using electrophysiological recordings (Fig. 4, D to I). The conclusions were the same as for morphological synapse measurements. Specifically, chronic BACE1 inhibition decreased (~20%) the frequency of mEPSCs in WT neurons, whereas, as described above, the Swedish mutation in *APP* increased (30 to 40%) the mEPSC frequency. BACE1 inhibition of Swedish-mutant neurons markedly suppressed the mEPSC frequency (40 to 60% decrease), such that it was similar to the mEPSC frequency observed in BACE1-inhibited WT neurons (Fig. 4, D to I). None of these manipulations altered the mEPSC amplitude. Together, these results indicate that BACE1 inhibition reduces the synapse density in a manner dependent on APP that occludes the effect of the *APP*-Swedish mutation.

### Conditional deletion of *APP* impairs synaptic function

**Fig. 5.**
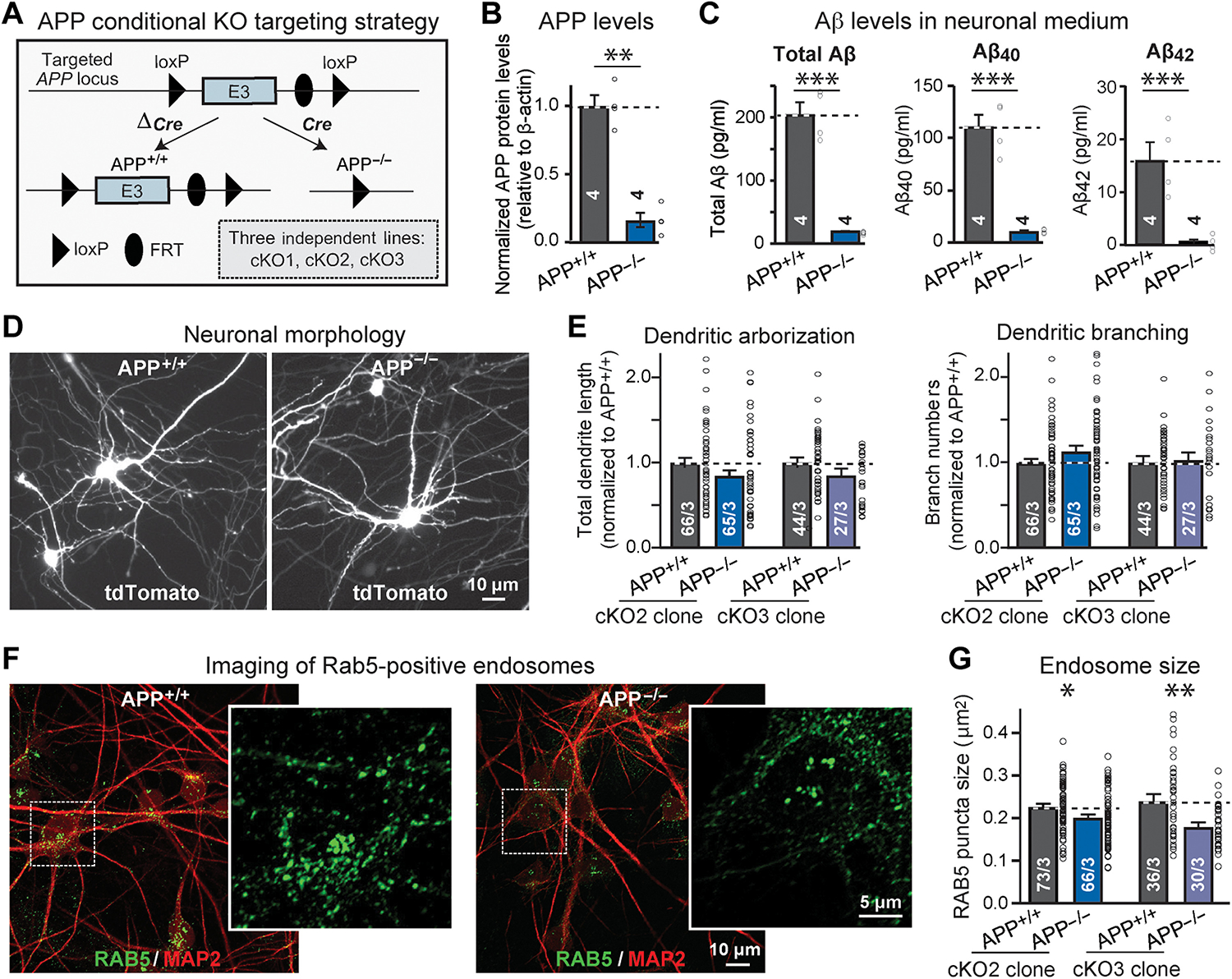
Generation and characterization of isogenic human *APP*^+/+^ and *APP*^−/−^ neurons derived from the same conditional *APP* knockout stem cell subclones. (A) Design of the conditional *APP* knockout allele. (B) Immunoblotting analysis of APP protein to validate the removal of *APP* alleles in human neurons with *APP* conditionally deleted. The remaining APP protein is derived, at least in part, from the cocultured mouse glia that also express APP, albeit at lower amounts. (C) Validation of the *APP* deletion in human *APP*^−/−^ neurons measured by Aβ peptides ELISA on supernatants harvested at 17 days after neuronal induction with Ngn2. (D and E) Dendritic arborization of human *APP*^+/+^ and *APP*^−/−^ neurons derived from the same APP conditional knockout stem cell clones. [(D) representative images of sparsely transfected human *APP*^+/+^ and *APP*^−/−^ neurons expressing tdTomato; (E) quantification of the total dendrite length and number of branches of *APP*^+/+^ and *APP*^−/−^ neurons from two independent mutant hES cells clones (cKO2 and cKO3)]. (F and G) Assessment of the Rab5-positive endosomes in *APP*^−/−^ neurons and their isogenic *APP*^+/+^ controls. [(F) representative images of *APP*^+/+^ and *APP*^−/−^ neurons immunolabeled for Rab5 (green) and MAP2 (red); (G) quantification of the size of Rab5-positive endosomes (insets, expanded views of endosomes) in human *APP*^+/+^ and *APP*^−/−^ neurons generated from two independent mutant hES cells clones (cKO2 and cKO3)]. All summary graphs show means ± SEM [number of experiments (B and C) or cells/experiments (E and G) are indicated in bars]; statistical significance was assessed by Student’s t test, with *P < 0.05, **P < 0.01, and ***P < 0.001. Nonsignificant comparisons are not indicated. Additional data are shown in fig. S7.

The results of the experiments with Swedish-mutant neurons and BACE1 inhibition suggest that APP cleavage serves to promote synapses. If so, then deletion of *APP* should decrease synaptic function. To test this prediction, we introduced a conditional loss-of-function mutation into the *APP* gene in hES cells (Fig. 5A). Different from the conditionally *APP*-Swedish mutation (Fig. 1A), the strategy for generating conditional deletions of *APP* is much simpler. We introduced loxP sites that flank exon 3 of the *APP* gene, which lead to a deletion of APP expression upon Cre-mediated excision (Fig. 5A and fig. S6, A to C). However, in contrast to engineering of the heterozygous Swedish mutation, we had to target both alleles of the *APP* gene to create a complete loss of APP function.

Conditional deletion of *APP* in human neurons greatly lowered (>80%) APP expression (Fig. 5B and fig. S6, D and E). Because Cre virus infection rate of neurons was close to 100%, the remaining APP protein is likely derived from cocultured nonmutant glia. As expected, the APP deletion also severely suppressed Aβ secretion (>90%) (Fig. 5C). The *APP* deletion had no effect on neuronal survival or dendritic arborization (Fig. 5, D and E, and fig. S7, A to E). However, the *APP* deletion caused a decrease (10 to 25%) in the size of Rab5-positive endosomes (Fig. 5, F and G, and fig. S7F). Thus, there is a matching increase in the size of Rab5-positive endosomes in *APP*-Swedish–mutant human neurons (Fig. 2, C and D) but a decrease in *APP*-deficient neurons (Fig. 5, F and G).

**Fig. 6.**
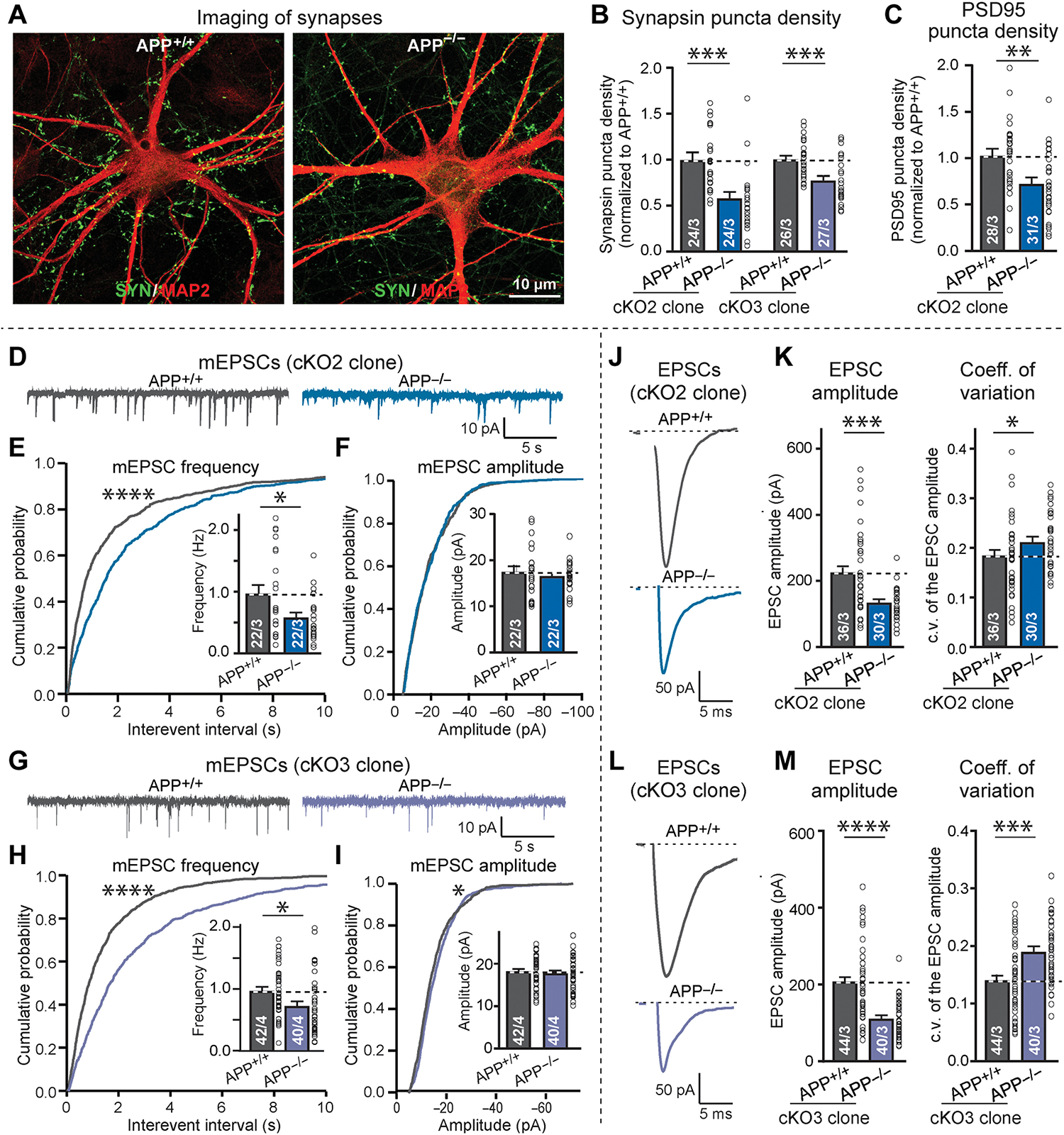
Synapse density and synaptic transmission in human *APP*^+/+^ and *APP*^−/−^ neurons. (A to C) Quantification of synapsin- and PSD95-positive synaptic puncta in human *APP*^+/+^ and *APP*^−/−^ neurons derived from the same *APP* conditional knockout stem cell clones [(A) representative images of human *APP*^+/+^ and *APP*^−/−^ neurons immunolabeled for synapsin (green) and MAP2 (red); (B) quantification of the density of synapsin-positive puncta in *APP*^+/+^ and *APP*^−/−^ neurons derived from two independent mutant hES cells clones (cKO2 and cKO3); (C) quantification of the density of PSD95-positive puncta in *APP*^+/+^ and *APP*^−/−^ neurons generated from cKO2 hES cells]. For representative images, see fig. S7 (G to I). (D to I) Assessment of the frequency and amplitude of mEPSCs in *APP*^+/+^ and *APP*^−/−^ neurons derived from cKO2 (D to F) and cKO3 clones (G to I) [(D and G) representative traces; (E and H) cumulative probability plots of the interevent times, and summary graph of the mEPSC frequency (inset); (F and I) cumulative probability plots and summary graphs (inset) of mEPSC amplitudes]. (J to M) Assessment of the amplitude and coefficient of variation of the evoked EPSCs measured on isogenic *APP*^+/+^ and *APP*^−/−^ human neurons derived from cKO2 (J and K) and cKO3 clones (L and M) [(J and L) representative traces; (K and M) summary graphs of EPSC amplitudes (left) and coefficients of variation (right)]. All data were obtained from human neurons at 5 weeks after neuronal induction with Ngn2; additional data are shown in fig. S7. All summary graphs show means ± SEM (cells per experiments are indicated in bars); statistical significance was assessed by Student’s t test (bar graphs) or KS test (cumulative probability plots), with *P < 0.05, **P < 0.01, ***P < 0.001, and ****P < 0.0001. Nonsignificant comparisons are not indicated.

The *APP* deletion caused a robust decrease (20 to 35%) in synapse density, as measured by immunocytochemistry for either synapsin or PSD95 (Fig. 6, A to C, and fig. S7, G to I). This decrease was detected in two independent clones on an isogenic background. Electrophysiological recordings, analyzing neurons derived from two independent clones, demonstrated that the *APP* deletion caused a decline in mEPSC frequency (20 to 30%) without affecting the mEPSC amplitude or intrinsic electrical properties (Fig. 6, D to I, and fig. S7J). Last, the *APP* deletion suppressed the amplitude of evoked EPSCs (30 to 40% decrease), which was at least in part likely due to a decrease in release probability as judged by the increase (10 to 25%) in the coefficient of variation (Fig. 6, J to M). Thus, the deletion and Swedish mutation of *APP* induce mirror-image phenotypes: The *APP* deletion suppresses synapse numbers and synaptic function, but the *APP*-Swedish mutation enhances them. In the set of experiments described in the following, we explored how these phenotypes are produced.

### Exogenous Aβ promotes synapse formation in human neurons

**Fig. 7.**
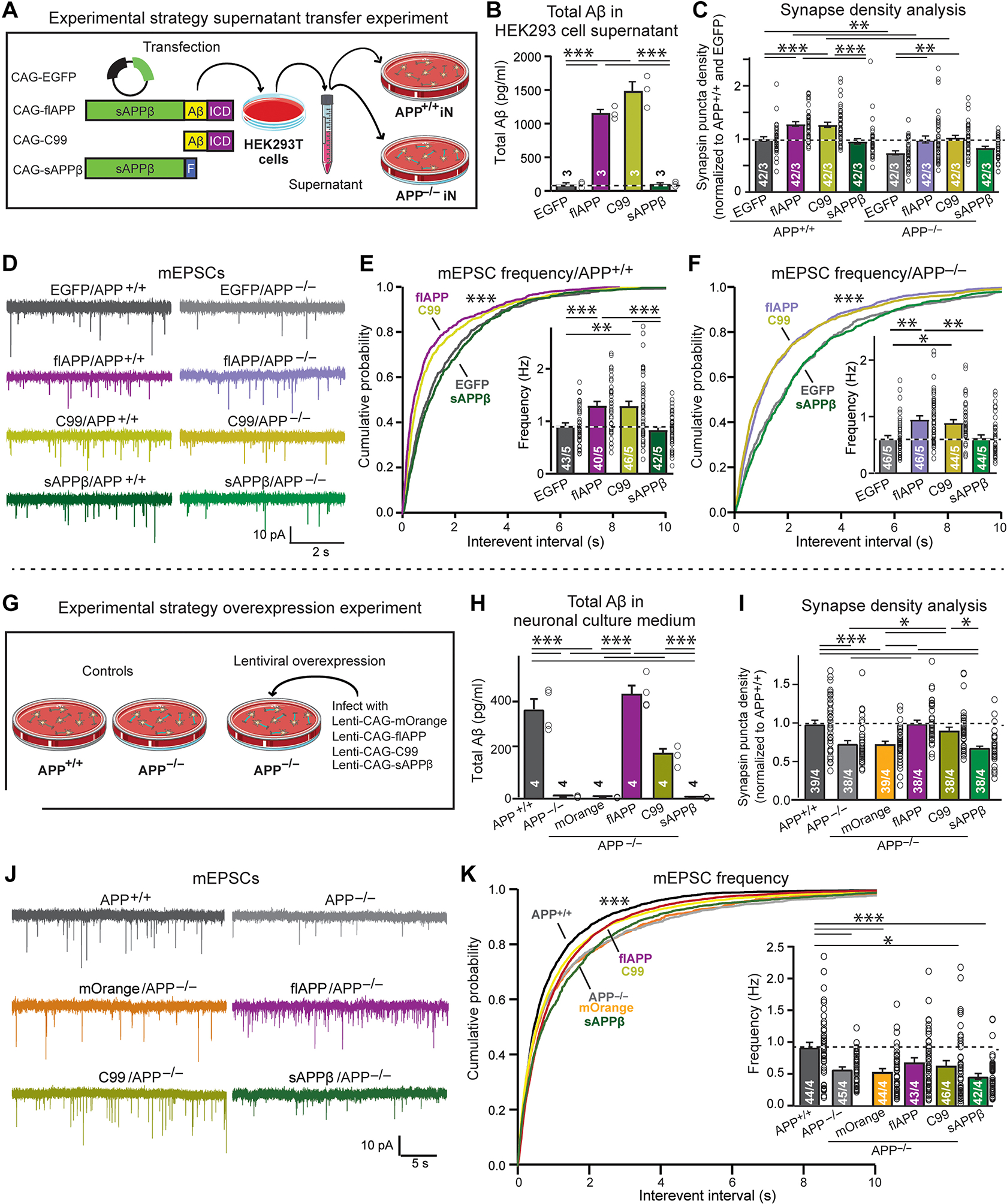
The effect of modest elevations of Aβ on synapse formation in human neurons. (A) Experimental strategy for (B) to (F). The supernatant of HEK293T cells expressing EGFP (control), full-length APP (flAPP), or the C99 and sAPPβ fragments of APP was added to human *APP*^+/+^ and *APP*^−/−^ neurons 7 days after neuronal Ngn2 induction; neurons were analyzed 4 weeks afterwards. (B) Total Aβ peptides measured by ELISA in supernatants of HEK293T cells expressing EGFP, full-length APP, C99, and sAPPβ. (C) Quantification of synapse density measured by synapsin immunocytochemistry on human *APP*^+/+^ and *APP*^−/−^ neurons treated with indicated supernatants from (A). For representative images, see fig. S8F. (D to F) Assessment of the mEPSC frequency in human *APP*^+/+^ and *APP*^−/−^ neurons treated with indicated supernatants from (A). [(D) Representative mEPSC traces; (E and F) cumulative probability plots of mEPSC interevent intervals (insets, summary graphs of the mEPSC frequency) from *APP*^+/+^ (E) and *APP*^−/−^ (F) neurons]. (G) Experimental strategy for (H) to (K). Human *APP*^−/−^ neurons were infected with lentiviruses encoding mOrange (control), full-length APP (flAPP), or the C99 and sAPPβ fragments of APP 4 days after neuronal Ngn2 induction; neurons were analyzed 5 weeks after neuronal induction. Uninfected *APP*^+/+^ and *APP*^−/−^ neurons were used as further controls in (H) to (K). (H) Total Aβ peptides in supernatants of human *APP*^−/−^ neurons expressing EGFP, full-length APP, C99, and sAPPβ. Values were measured by ELISA at 17 days after neuronal Ngn2 induction. (I) Quantification of synapse density by synapsin immunocytochemistry on human *APP*^−/−^ neurons expressing EGFP, full-length APP, C99, and sAPPβ. For representative images, see fig. S9F. (J and K) Assessment of the mEPSC frequency on human *APP*^−/−^ neurons expressing EGFP, full-length APP, C99, and sAPPβ. [(J) Representative mEPSC traces; (K) cumulative probability plot of mEPSC interevent intervals and summary graph of the mEPSC frequency(inset)]. Additional data are shown in figs. S8 and S9. All summary graphs show means ± SEM [number of experiments (B and H) or of images or cells/experiments (all other graphs) are indicated in bars]; statistical significance was assessed by one-way ANOVA (B, H, I, and K) or two-way ANOVA (C, E, and F) with post hoc corrections, or by KS test (cumulative probability plots), with *P < 0.05, **P < 0.01, and ***P < 0.001. Nonsignificant comparisons are not indicated.

Our data indicate that a BACE1-dependent cleavage products of APP regulate synapses. Three APP cleavage products are increased by the *APP*-Swedish mutation and suppressed by the *APP* deletion: the secreted extracellular APPs fragment after BACE1 cleavage (sAPPβ), the remaining, transient C-terminal APP fragment after BACE1 cleavage (C99, also referred to as CTFβ), and the secreted Aβ peptide released from the C99 fragment by γ-secretases. To identify which of these products mediates the synaptic effects of APP cleavage by BACE1, we transfected human embryonic kidney (HEK) 293T cells with full-length APP (positive control), sAPPβ, and C99, using enhanced green fluorescent protein (EGFP)–only transfections as a negative control (Fig. 7A). The supernatants from HEK293T cells expressing full-length APP or C99, but not from HEK293T cells expressing sAPPβ or EGFP only, contained elevated Aβ as expected (Fig. 7B), whereas the supernatants from HEK293T cells expressing full-length APP or sAPPβ, but not from HEK293T cells expressing C99 or EGFP only, contained higher amounts of sAPPβ (fig. S8A). We added the supernatants from the transfected HEK293T cells to matching WT and *APP*-deleted human neurons and analyzed these neurons using morphological and electrophysiological assays (Fig. 7A). The amount of added supernatant produced a final Aβ concentration in the medium of the neurons (~0.4 to 0.5 ng/ml) comparable to that observed in the medium of neurons carrying the *APP*-Swedish mutation. Immunoblotting demonstrated that Aβ in fresh HEK293T cell supernatants, as added to the neuronal culture medium, was largely monomeric, whereas upon storage, the Aβ became oligomeric (fig. S8C).

Supernatants from HEK293T cells did not change the size of Rab5-positive endosomes compared to the supernatant from EGFP control in both WT and *APP*-deficient human neurons, although there was a trend for an increase in size in neurons treated with supernatants from HEK293T cells expressing full-length APP or C99 (fig. S8, D and E). However, supernatants from HEK293T cells expressing full-length APP or C99, but not the other supernatants, increased (~25%) the density of synapsin-positive puncta in WT human neurons without changing their size (Fig. 7C and fig. S8, F and G). As before, *APP*-deficient neurons exhibited a decreased synapse density (~20%), but, again, the full-length APP and C99 supernatants increased the synapse density (~25%). An almost identical pattern of responses was detected when we recorded mEPSCs, with the full-length APP and C99 supernatants robustly increasing the mEPSC frequency (~30%) in both WT and *APP*-deficient human neurons without changing the mEPSC amplitude (Fig. 7, D to F, and fig. S8, H and I). Together, these results can be explained by an effect of Aβ because Aβ is very likely the only product that is present in the full-length APP and C99 supernatants but lacking in the sAPPβ and control supernatants.

A potential role of Aβ in promoting synaptic function is unexpected given the vast literature suggesting the opposite, albeit on the basis of experiments applying rather high Aβ concentrations to rodent neurons (52–54). We also find that direct applications of Aβ to human neurons are highly toxic. To definitively establish a role for Aβ in synapse formation, we sought to test its role using a second, complementary approach. We generated matching WT and *APP*-deficient human neurons and used lentiviruses to express in the *APP*-deficient neurons either mOrange as a negative control or full-length *APP*, sAPPβ, or C99 (Fig. 7G). Measurements of Aβ and sAPPβ secreted into the medium demonstrated that only expression of full-length APP and C99 (but not of mOrange or sAPPβ) resulted in physiological Aβ production, whereas conversely expression of full-length APP and sAPPβ (but not of mOrange and C99) induced secretion of physiological sAPPβ (Fig. 7H and fig. S9, A to C).

In contrast to the supernatant experiments, the decreased size of Rab5-positive endosomes (~25%) in *APP*-deleted neurons was fully reversed by expression of full-length APP or C99 but not by expression of mOrange or sAPPβ (fig. S9, D and E). Expression of full-length APP and C99, but not of mOrange or sAPPβ, fully restored synapse numbers that were suppressed (~25%) by the *APP* deletion (Fig. 7I and fig. S9, F and G). Furthermore, recordings of mEPSCs demonstrated that expression of full-length APP and C99, but not of mOrange or sAPPβ, rescued most of the decrease in mEPSC frequency induced by the deletion of *APP*, although the rescue achieved statistical significance only in the cumulative distributions (p < 0.0001) and not in the means (Fig. 7, J and K, and fig. S9H). Thus, Aβ at physiological amount reverses the synaptic phenotypes of *APP*-deficient human neurons.

## DISCUSSION

Our results suggest that contrary to currently prevailing concepts, Aβ at physiological concentrations is not neurotoxic and synaptotoxic but supports synapse function in human neurons. We used a rigorous genetic approach with isogenic controls and four independent approaches to arrive at this conclusion: analysis of human neurons carrying the *APP*-Swedish mutation that causes familial AD, demonstration of the role of BACE1 using a well-validated BACE1 inhibitor in WT and *APP*-mutant human neurons, analysis of human neurons carrying a deletion of *APP*, and measurements of the effects of increasing Aβ directly. These experiments suggest that, physiologically, APP promotes synapse formation in human neurons at least in part via release of Aβ peptides.

Why did previous studies arrive at a different conclusion? Studies in human and mouse neurons often involved high concentrations of Aβ and were performed under conditions that made control for genetic background and for clonal variation difficult (41–43). It is possible that oligomeric Aβ is neurotoxic but not normally produced by neurons (55), whereas monomeric Aβ may be synaptogenic. This would agree with the delayed development of AD in patients, suggesting that secreted endogenous Aβ may only become oligomeric as it accumulates during a person’s lifetime, similar to what is observed in vitro. Moreover, recombinant or overexpressed Aβ is more likely oligomeric than endogenous Aβ, thereby enhancing its toxicity. Our results confirm the previously observed increase in the size of Rab5-positive endosomes in human neurons with *APP* mutations that are associated with familial AD (38) but reveal that relative to the synaptic phenotype produced by the *APP*-Swedish mutation, the endosomal change is rather small. Given the increase in endosome size and synapse numbers in *APP*-Swedish–mutant and *APP*-deficient neurons, further research is needed to reveal the relation of endosomes to synapse formation in neurons.

Using cultured human neurons, we found that the *APP*-Swedish mutation increases synaptic function, which is quite counterintuitive. In the same vein, we and others observed an increase in synapse numbers with apolipoprotein E4 (ApoE4) similar to that detected here with Aβ released from APP, which is also puzzling in suggesting that ApoE4, the most important genetic risk factor for sporadic AD, predisposes to AD but enhances instead of suppressing synapse formation (44, 56, 57). ApoE4 also increases the size of endosomes in human neurons (44), similar to endogenous Aβ in our experiments. Neither of these findings was anticipated, nor can we offer a ready explanation for why a genetic predisposition to AD should be enhancing synapse numbers and endosome sizes. A key to reconciling the synaptogenic and neuropathological effects of Aβ and ApoE4 may be the timelines involved. Whereas synapse formation occurs in hours, AD pathogenesis operates over decades. The relation of synapse formation to synaptic degeneration is unclear, especially because synapse formation proceeds throughout life, and many synapses are short lived (58). Although unexpected, our findings offer a potential explanation for the consistent clinical finding that pharmacological suppression of Aβ by BACE1 inhibition leads to cognitive dysfunction (9–10), which could be due to a loss of synapses. We find this hypothesis intriguing because the BACE1 inhibition–mediated cognitive decline in patients with AD is reversible (59). Again, these questions will need to be addressed with new experimental approaches—time will tell.

Our study has important limitations. All of our work was performed with cultured excitatory neurons with a relatively, although not completely, homogeneous composition. In a living brain, many different types of neurons are connected into vast communicating networks that are supported by active types of glia, whereas cultures represent a less complex two-dimensional preparation. However, cultures reduce the environmental complexity and may serve as an appropriate model for the effects of single molecules/genes in human neuronal diseases. It seems likely that the fundamental properties of the basic units of circuits, such as their synapses, will be similarly affected by the *APP* mutations we analyzed in two- or three-dimensional states, but the effect of these mutations is difficult to analyze in a complex brain context. Different types of neurons, especially excitatory and inhibitory neurons, may be affected differentially by the *APP* mutations. Moreover, the impact of the mutations we examined on circuits is difficult to predict, not only because we only studied a restricted range of excitatory neurons but also because we did not examine these neurons in a real brain with all of its complexity. However, human neurons and their synapses are not readily amenable for studies in an intact brain. Although organoids have provided enormous progress in this regard, neuronal maturation in organoids is generally more limited than in two-dimensional cultures and little synaptic circuit analyses have been possible.

Mouse models of AD that involve transgenic expression of mutant APP have produced diverse phenotypes, with some results agreeing well with the present findings. Transgenic mice expressing APP with AD-associated mutations exhibit hyperexcitability, consistent with increased synaptic function (60). In young mice, the *APP*-Swedish mutation increases the spine density as a proxy for synapse density, but in aging mice, it decreases the spine density (61). Similarly, recent studies suggest that elevated Aβ in mice enhance synaptic protein expression (62), whereas the APP knockout may decrease spine and synapse numbers (63). Although more detailed studies on synapses in mutant mice will be required to conclude how general these findings are, they are congruent with the notion proposed here that Aβ is a pro-synaptogenic factor in young neurons at physiological concentrations.

In summary, we show here that in human neurons, the Swedish mutation in *APP* that enhances Aβ secretion promotes synapse formation, whereas deletion of *APP* or pharmacological BACE1 inhibition suppresses synapse formation. Taken together, these data provide a direct link of AD pathogenesis to synapse function that may help in designing AD prevention strategies.

## MATERIALS AND METHODS

### Study Design

This study was designed to thoroughly characterize *APP*-Swedish mutant and *APP*-deficient human neurons, particularly on synapse formation and synapse activity. For this purpose, we made hES/iPS cell lines carrying conditional *APP*-Swedish mutation or conditional *APP*-deficient alleles, and generated *APP*-Swedish mutant and *APP*-deficient human neurons along with their isogenic controls, with Cre or Flp induced genome recombination. Moreover, BACE1 inhibitor was applied to test whether the phenotype was caused by APP Cleavage in human neurons. In addition, we also elevated different APP cleavage products in human neuron medium to identify the one(s) elevating synapse formation. For all experiments, at least three biological replicates (independent experiments) were performed. All analyses were performed out in a ‘blinded’ fashion whereby the experimenter was unaware of the sample identity.

### Statistics

No statistical methods were used to predetermine sample size because effect sizes were unknown before experiments. Statistical significances for comparisons between two conditions were called with two-tailed t-test in Microsoft Excel or GraphPad Prism. For comparisons between more than two conditions, one-way ANOVA was performed with Tukey’s post hoc in GraphPad Prism. Interactions between two factors were tested with two-way ANOVA with Tukey’s post hoc in GraphPad Prism.

The Kolmogorov-Smirnov test was applied for cumulative curves. All data in bar graphs and summary plots are shown as means ± SEM of independent biological replicates in all figures. Numbers in bars represent number of cells/cultures analyzed, with statistical significance (∗ = p < 0.05, ∗∗ = p < 0.01, ∗∗∗ = p < 0.001 and ∗∗∗∗ = p < 0.0001).

See Supplementary Material section for detailed materials and methods.

## Supplementary Material

Table S1 Table S1. Individual subject-level data for statistics with n<20. (See supporting files)

supplemental material Fig. S1. Generation and verification of human ES and iPS cells with heterozygous knockin mutations in the *APP* gene that produces a conditionally Swedish-mutant *APP* allele.
Fig. S2. Characterization of induced human neurons derived from the human ES cell line H1.
Fig. S3. Further detailed characterizations of human neurons carrying *APP*-Swedish mutation, with the analysis data for additional clones that were only partly included in Fig. 1 & 2.
Fig. S4. Additional data on synapse formation and intrinsic electrical properties of *APP*^+/+^ and *APP*^Swe/+^ human neurons.
Fig. S5. Additional data demonstrating the effect of the BACE1 inhibitor LY2886721 (BACEi) on Aβ/sAPP secretion and on the relative concentration of APP mRNAs in human *APP*^+/+^ and *APP*^Swe/+^ neurons.
Fig. S6. Strategy for, and verification of, homozygous conditional KO mutations of *APP* in multiple engineered ES cell clones.
Fig. S7. Additional experiments characterizing human *APP*^−/−^ neurons compared to their genetically precisely matched *APP*^+/+^ controls that were derived from the same conditionally mutant parental ES cells line, including dendrite development, synapse puncta sizes, and intrinsic electrical properties.
Fig. S8. Assessments of the secretion and aggregation of Aβ and sAPPβ that were obtained in the supernatants of transfected HEK293T cells, and measurements of their effects on the size of Rab5-positive endosomes, size of synaptic puncta, and mEPSC amplitudes in isogenic *APP*^+/+^ and *APP*^−/−^ human neurons.
Fig. S9. Validation of the lentiviral expression of full-length APP (flAPP) or of the C99 and sAPPβ fragments of APP in HEK293T cells and human neurons, and measurements of the effects of the expression of full-length APP (flAPP) or of the C99 and sAPPβ fragments of APP on the size of Rab5-positive endosomes, size of synaptic puncta, and mEPSC amplitudes in human APP^−/−^ neurons compared to precisely matched *APP*^+/+^ controls.

## Data Availability

All data are available in the main text or the supplementary materials.
