# Supplementary material for: Synaptogenic effect of APP-Swedish mutation in familial Alzheimer’s disease
Source: Sci Transl Med. Author manuscript; Available in PMC 2023 Feb 2. (PMC9894682; doi:10.1126/scitranslmed.abn9380)
Supplement: supplemental material — Fig. S1. Generation and verification of human ES and iPS cells with heterozygous knockin mutations in the APP gene that produces a conditionally Swedish-mutant APP allele. Fig. S2. Characterization of induced human neurons derived from the human ES cell line H1. Fig. S3. Further detailed characterizations of human neurons carrying APP-Swedish mutation, with the analysis data for additional clones that were only partly included in Fig. 1 & 2. Fig. S4. Additional data on synapse formation and intrinsic electrical properties of APP+/+ and APPSwe/+ human neurons. Fig. S5. Additional data demonstrating the effect of the BACE1 inhibitor LY2886721 (BACEi) on Aβ/sAPP secretion and on the relative concentration of APP mRNAs in human APP+/+ and APPSwe/+ neurons. Fig. S6. Strategy for, and verification of, homozygous conditional KO mutations of APP in multiple engineered ES cell clones. Fig. S7. Additional experiments characterizing human APP−/− neurons compared to their genetically precisely matched APP+/+ controls that were derived from the same conditionally mutant parental ES cells line, including dendrite development, synapse puncta sizes, and intrinsic electrical properties. Fig. S8. Assessments of the secretion and aggregation of Aβ and sAPPβ that were obtained in the supernatants of transfected HEK293T cells, and measurements of their effects on the size of Rab5-positive endosomes, size of synaptic puncta, and mEPSC amplitudes in isogenic APP+/+ and APP−/− human neurons. Fig. S9. Validation of the lentiviral expression of full-length APP (flAPP) or of the C99 and sAPPβ fragments of APP in HEK293T cells and human neurons, and measurements of the effects of the expression of full-length APP (flAPP) or of the C99 and sAPPβ fragments of APP on the size of Rab5-positive endosomes, size of synaptic puncta, and mEPSC amplitudes in human APP−/− neurons compared to precisely matched APP+/+ controls. [file NIHMS1863569-supplement-supplemental_material.pdf]

Science Translational Medicine
AAAS

Supplementary Materials for

Synaptogenic effect of *APP*-Swedish mutation in familial Alzheimer's disease

Bo Zhou *et al.*

Corresponding author: Marius Wernig, wernig@stanford.edu; Thomas C. Südhof, tcs1@stanford.edu

Sci. Transl. Med. **14**, eabn9380 (2022)
DOI: 10.1126/scitranslmed.abn9380

The PDF file includes:

Materials and Methods
Figs. S1 to S9
Legend for table S1
Table S2
References (*45*, *64–66*)

Other Supplementary Material for this manuscript includes the following:

Table S1

Supplementary materials and methods

Experimental procedures

Cell Culture

Human H1 ESCs and induced pluripotent stem (iPS) cells were maintained in mTeSR1 medium (Stem Cell Technologies) or StemFlex (Thermo Fisher) and used at intermediate passage numbers as described (45, 56, 57, 64-66) for genome targeting and generating human induced neuronal (iN) cells. Mouse glial cells were isolated from the forebrain of P0-P3 wild-type CD1 male and female pups, digested into single cells by Papain (Worthington), and maintained in DMEM-based medium with 5% FBS as described earlier (*57, 64*). Upon passaging for 1-2 times, glial cells were replated on matrigel-coated coverslips for co-culturing with iN cells. All animal experiments were approved by the Stanford animal use committees (IACUC).

Virus generation

Lentiviruses were produced as described (*64-66*). Generally, lentiviral vectors were co-transfected into HEK293T cells (ATCC) with three helper plasmids (pRSV-REV, pMDLg/pRRE and vesicular stomatitis virus G protein expression vector) using Polyethylenimine (PEI). Lentiviruses were harvested along with the supernatants 30 h and 46 h after transfection. Lentiviral particles were ultra-centrifuged, re-suspended in DMEM with sucrose, aliquoted and stored at −80°C.

Targeting AAVs were produced from HEK293T cells by co-transfection of pAAV, helper adenovirus type 5 (pAd5) and AAV-DJ61 using PEI as described (64). Cells were harvested 72 h after transfection in PBS with 1 mM EDTA followed by three freezing thawing cycles. After incubating at 37°C for 1 h with 250 units/ ml of Benzonase, the cytoplasm mixture was centrifuged at 2,000 x g for 15 min to remove cell debris. The supernatant was harvested, aliquoted and stored at -80°C.

Genome Targeting

APP conditional Swedish-mutant and *APP* conditional knockout (KO) ES and iPS cell clones were obtained by recombinant adenoassociated virus (rAAV) mediated homologous recombination. For *APP* conditional Swedish-mutant clones, the rAAV targeting vector was designed to carry two homology arms separated by the mutant *APP* Exon16 floxed by LoxP and FRT, a splice acceptor (SA) followed by an internal ribosomal entry site (IRES) and the Neomycin resistance gene (NEO) with a polyadenylation signal (PA), and the wild-type *APP* Exon16 floxed by LoxP and FRT (Fig. 1A, Fig. S1). Neomycin-resistant clones were picked after rAAV infection. Clones with correct homologous recombination were then identified by PCR with one primer external to the homology arms and the other primer sitting on the LoxP sites. The ones with correct size of PCR products were selected. Next, to verify the homogeneity of the positive clones, PCR products of *APP* Exon16 from each clone's genome DNA were TOPO-cloned followed by Sanger sequencing. Clones were selected for further experiments only when ~1/3 of the PCR amplicons contain the Swedish mutation.

For *APP* conditional KO clones, the two *APP* alleles of H1 cells were targeted sequentially by an rAAV carrying two homology arms separated by wild type Exon 3 floxed by LoxP and FRT, a SA-IRES-NEO-PA, FRT and LoxP (Fig. S6A). After the first round of targeting, Neomycin-resistant clones were picked and screened with PCR primers spanning the homology arms as described above. Afterwards, the SA-IRES-NEO-PA cassette was removed by transient FlpE recombinase expression. The cells were then targeted by the same rAAV to induce homology recombination on the other *APP* allele, followed by SA-IRES-NEO-PA cassette removal with FlpE recombinase expression. PCR and immunoblot were applied to verify the positive clones.

Generation of iN cells

Ngn2-iN cells were generated as described (45, 57, 64-66). ESCs or iPS cells were treated with Accutase, plated as dissociated single cells on Matrigel-coated plates, and infected with Ngn2 and rtTA lentiviruses in mTeSR1 or StemFlex medium. The next day (DIV1), the culture medium was replaced with DMEM/F12 with addition of N2, NEAA, 1 mM Insulin and 2 μg/ml doxycycline to induce Ngn2 expression. On DIV2 and 3, puromycin was used to select infected cells. Cre/ ΔCre/ Flp lentiviruses were added on DIV4 to induce recombination. On DIV 6, iN cells were dissociated using Accutase and plated on mouse glial cells-plated coverslips at 150-200 K cells/well in 24-well plate in the Neurobasal medium with B27, GlutaMax, Sodium Pyruvate, and 2% FBS. Culture medium was half

changed every 6 days. Supernatants were harvested for ELISA on DIV17 unless indicated. For other assays, iN cells were harvested 5 weeks after doxycycline treatment.

For BACEi treatment, cells were treated with 2 µM LY2886721 chronically from DIV7 until harvesting. For the supernatant-treatment experiment, the CAX-driven constructs were transfected into HEK293T cells using PEI. Supernatants from the transfected HEK293T cultures were harvested 3-day and 6-day after transfection and kept at 4ºC for no longer than 1 month. The supernatants were added to iN cultures (1:3) chronically from DIV7 until harvesting. For the lentivirus-overexpression experiment, the iN cells were infected with lentiviruses along with Cre/ΔCre on DIV4.

ELISA

Enzyme-linked immunosorbent assay of Total Aβ, Aβ40, Aβ42, sAPPα sAPPβ-wt and sAPPβ-swe from culture supernatants (1:3-1:5 dilution), and of total Tau and pS396-Tau from cell lysates (1:100 dilution). All measurements were performed using ELISA kits listed in Key Resources Table according to the manufacturer's instructions.

Gene Expression Analyses

RNA was isolated using Trizol (Thermo Fisher Scientific) followed by Direct-zol RNA Kits (Zymo Research) with DNaseI. To measure the relative mRNA concentrations of interest, quantitative RT-PCR measurements were performed using TaqMan probes (Integrated DNA Technologies) with VeriQuest Probe One-Step qRT-PCR Master Mix (Affymetrix) on an 7900HT or QuantStudio3 (Applied Biosystems). All values presented were normalized to Gapdh or β-actin.

Analysis of dendrites

Mature iN cells were sparsely transfected with FCW-EGFP or FCW-tdTomato plasmid using Calcium Phosphate. Two days after transfection, cells were fixed and imaged under a Zeiss digital camera, with a 20x objective. Images were reconstructed using the MetaMorph neurite application, scoring for total dendrite outgrowths, number of processes, number of branch points, and cell body areas.

Immunofluorescence analysis

Immunofluorescence was performed on mature iN cells using antibodies against MAP2, Rab5, Synapsin-1 and PSD95. Confocal Z-stacked images were acquired with a Zeiss LSM710 laser-scanning confocal microscope using a 63x 1.4 NA oil objective. The stacked images were converted to maximal projection images and analyzed using ImageJ software.

For synapse density analysis, the numbers of Synapsin-1 or PSD95 puncta were normalized over the total MAP2 positive areas, with cell bodies masked off. For Rab5 puncta analysis, each iN cell body was masked based on MAP2 signal to quantify the average Rab5 puncta size in individual iN cell bodies.

Immunoblotting experiments

Total proteins were extracted from iN cells co-cultured on mouse glia 5 weeks after neuronal induction unless otherwise noted using RIPA buffer (50 mM Tris-Cl pH 8.0, 1mM EDTA, 1% Triton X-100, 0.1% Sodium deoxycholate, 0.1% SDS, 140 mM NaCl and 5% glycerol). After mixing with loading buffer, the protein samples were loaded on 4-20% TGX™ precast protein gels (Biorad) and transferred to 0.2 µm Nitrocellulose membranes via Trans-Blot Turbo transfer system (Biorad). The membranes were then blocked in TBST with 5% BSA, and incubated with primary antibodies overnight. Fluorescent secondary antibodies were applied for imaging and quantification on Odyssey DLx (LI-COR).

Electrophysiology

Whole-cell voltage-clamp recordings were performed on iN cells at D35-40, with 3–3.5 M borosilicate patch pipettes filled with an internal solution containing the following (in mM): 135 CsMeSO3, 8 NaCl, 10 HEPES, 0.25 EGTA, 4 MgATP, 0.3, Na_3GTP, 2 $MgCl_2$, 5 Na-phosphocreatine, and 2 QX314 (pH adjusted to 7.30 with CsOH). Cells were held at -70 mV in a bath solution containing the following (in mM): 140 NaCl, 10 HEPES, 10 glucose, 5 KCl, 3 $CaCl_2$ and1 $MgCl_2$ (pH adjusted to 7.40 with NaOH). For mEPSC recordings, tetrodotoxin (TTX; 1 µM) and picrotoxin (50 µM) were added to the bath solution. All electrophysiological recordings were performed with Multiclamp 700B amplifiers (Molecular Devices) and analyzed using Clampfit 10.7 (Molecular Devices). Details of the electrophysiological recordings and analyses were described previously (56, *57*, *64*).

Supplementary figures and figure legends

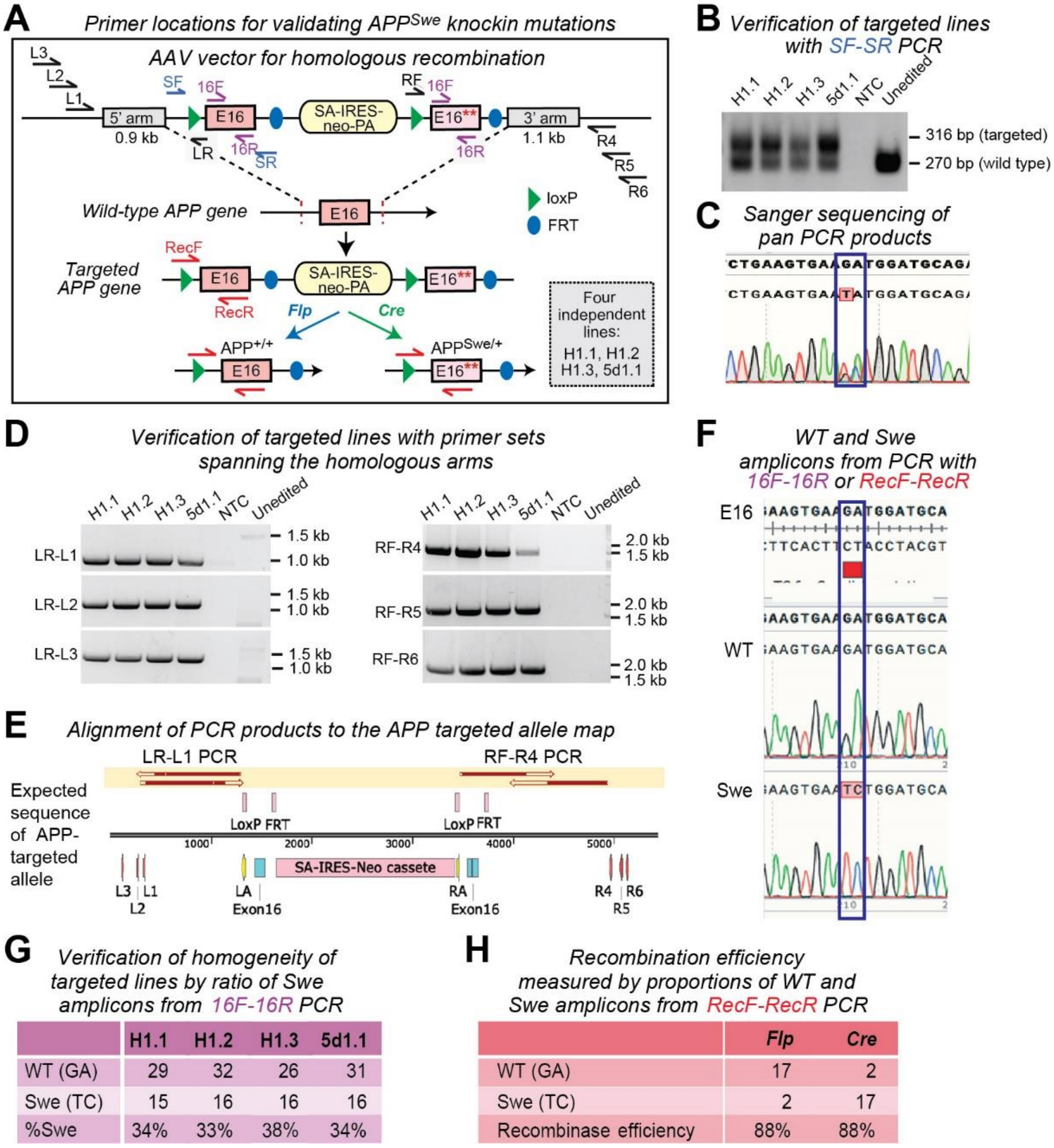

Fig. S1. Generation and verification of human ES and iPS cells with heterozygous knockin mutations in the *APP* gene that produces a conditionally Swedish-mutant *APP* allele. (A) Strategy for validating the heterozygous knockin Swedish-mutation in the *APP* gene using PCR. In the knockin allele, we inserted two copies of Exon 16 (E16), one WT and one carrying the Swedish mutation (red asterisks), and flanked the exons as well as the Neomycin resistance cassette with loxP and FRT sites as indicated. The knockin mutation was introduced into the endogenous *APP* locus of ES and iPS cells via AAV-mediated homologous recombination (*64*, *65*). The design of the knockin mutation is such that Flp recombination produces a wild-type *APP* allele ($APP^{+/+}$), whereas Cre recombination produces a Swedish-mutant *APP* allele ($APP^{Swe/+}$). Since the recombinases are introduced into cells after the induction of neuronal differentiation by Ngn2, the genotypes and culture conditions of the resulting $APP^{+/+}$ and $APP^{Swe/+}$ neurons are precisely matched. **(B)** PCR verification of correctly targeted ES and iPS cell clones with the primer set SF-SR that is shown in **A** ($APP^{+/+}$ PCR product, 270 bp; $APP^{Swe/+}$ Swedish-mutant PCR product, 316 bp). Clones produced in human H1 ES cells were labeled H1.1, H1.2 and H1.3, and the iPS cell-derived clone 5d1.1. NTC, no template control; Unedited, Untargeted H1 cells as negative controls. **(C)** Representative Sanger sequencing results of PCR

products of targeted clones; PCR products were obtained with the primer set 16F-16R. **(D)** PCR verification of targeted clones with one primer annealing at the loxP site (LR or RF), and the other primer external to the homologous arms (Primers L1, L2, L3 for the left homologous arm, Primers R4, R5, R6 for the right homologous arm). The PCR reactions validate correct homologous recombination at both the 5' and 3' sides of the vector (H1.1, H1.2, H1.3, and 5d1.1, correctly targeted clones; NTC, no template control; Unedited, Untargeted H1 cells as negative controls). **(E)** Alignment of the sequences of the LR-L1 and RF-R4 PCR amplicons with the expected sequence of the targeted *APP* allele. **(F)** Representative Sanger sequencing results of $APP^{+/+}$ (upper panel, with GA) and $APP^{Swe/+}$ allele (Swe, lower panel, with TC), obtained in bacterial plasmids topo-cloned from 16F-16R or RecF-RecR PCR amplicons. **(G)** Verification of the homogeneity of targeted cell clones. PCR amplicons obtained with primers 16F-16R in a on targeted clones were Topo cloned, and ~50 single bacterial colonies were expanded for Sanger sequencing. From the sequencing results, ~1/3 of the PCR amplicons carried the Swedish mutation. Because the primers amplify both targeted and untargeted alleles, this result indicates the genomic homogeneity of all the 4 targeted stem cell clones. **(H)** Calculation of the efficiency of Flp and Cre recombination. PCR amplicons with RecF-RecR in a on Flp- or Cre-recombined cells of clone H1.2 were Topo cloned, and ~20 bacterial colonies were expanded for Sanger sequencing. The primers should only amplify the targeted but not untargeted allele. Thus, the recombination efficiency of the Flp and Cre virus were calculated as #(WT)/[#(WT)+#(Swe)] and #(Swe)/[#(WT)+#(Swe)], respectively, based on the frequency of each allele in the bacterial plasmids.

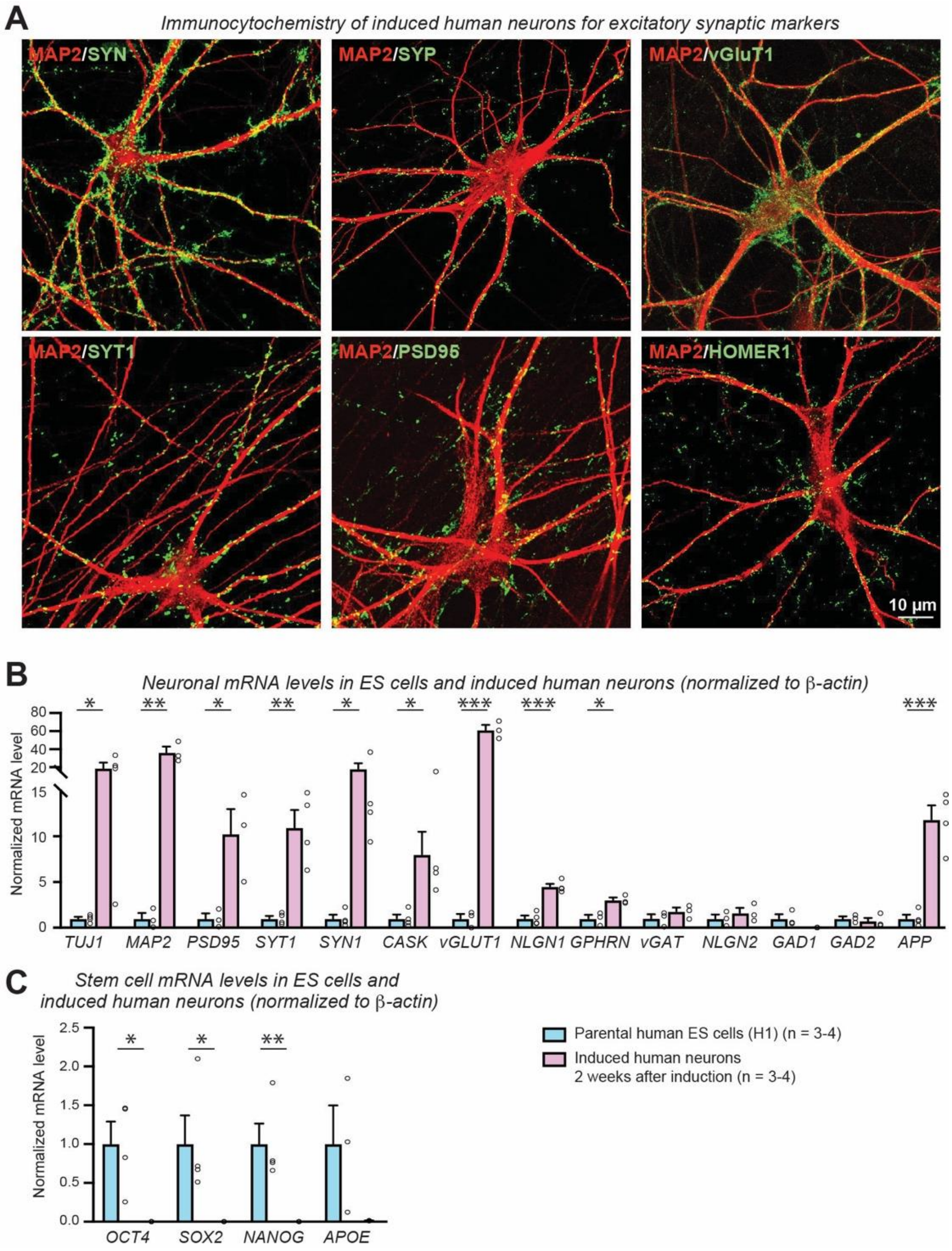

Fig. S2. Characterization of induced human neurons derived from the human ES cell line H1. **(A)** Immunocytochemistry for excitatory synaptic markers in human neurons derived from H1 ES cell line. Neurons were immunolabeled for Synapsin (SYN), Synaptophysin (SYP), vGLUT1, Synaptotagmin-1 (SYT1), PSD-95, and HOMER1, along with MAP2 as a dendritic marker. Immunocytochemistry was performed at 4 months (vGLUT1), 2 months (HOMER1), or 5 weeks (other markers) after neuronal induction. **(B & C)** Quantitative RT-PCR measuring human neuronal markers **(B)** and human embryonic stem cell markers **(C)** in human neurons 2 weeks after induction. All values were normalized to β-actin. All numerical data are means ± SEM. Statistical significance was assessed by Student's t-test, with*, $p<0.05$; **, $p<0.01$; *** and $p<0.001$. Non-significant comparisons are not indicated.

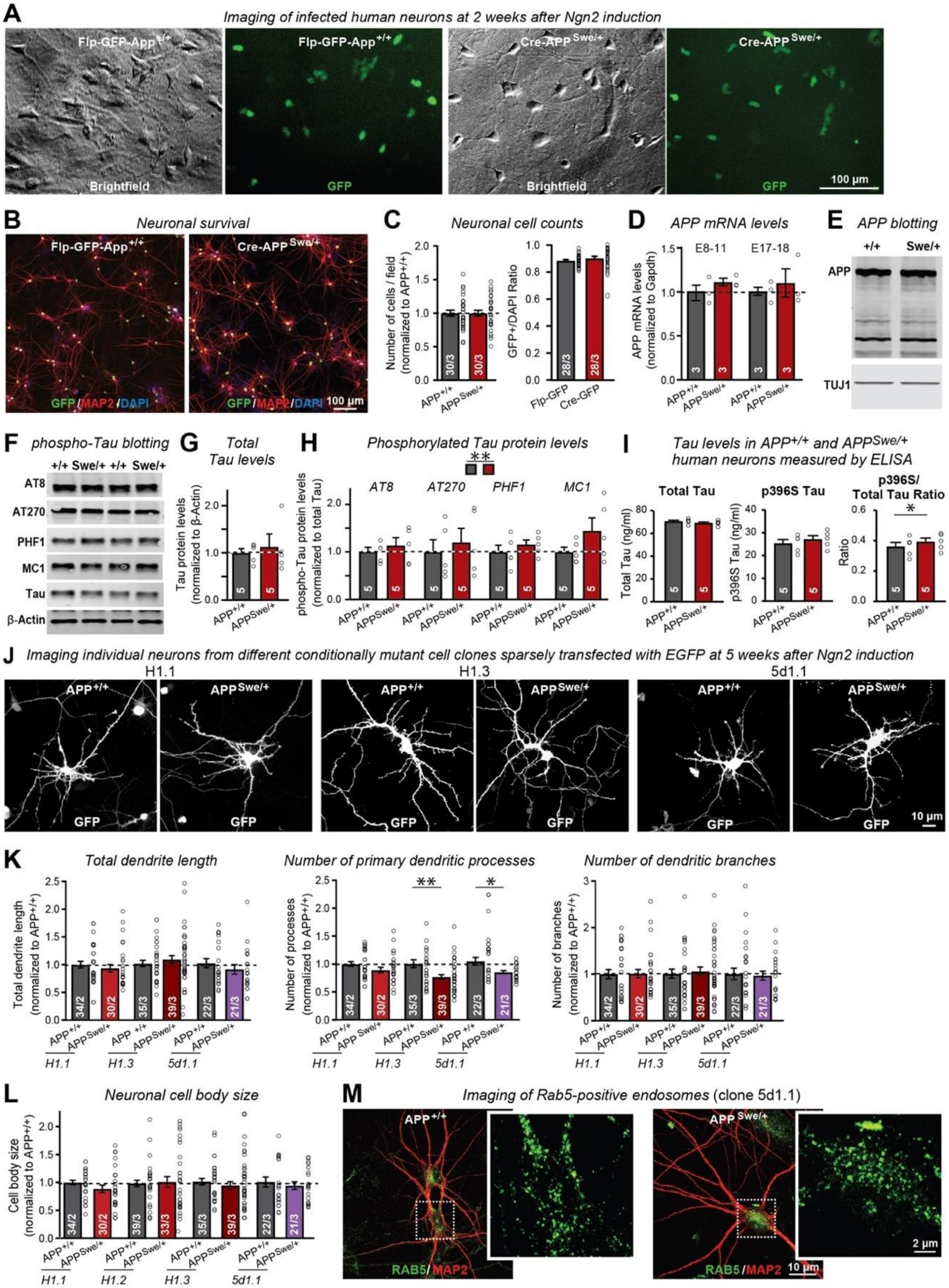

Fig. S3. Further detailed characterizations of human neurons carrying APP-Swedish mutation, with the analysis data for additional clones that were only partly included in Fig. 1 & 2. (A) Representative bright field and GFP fluorescent images of human neurons after Flp-GFP and Cre-GFP lentiviral infection 2 weeks after induction. Most human neurons in both conditions were GFP+, indicating the high infection efficiency of both viruses. **(B & C)**

Representative images (**B**) and quantification of MAP2+ cell numbers per field (**C-left**) and the percentage of infected MAP2+ cells (GFP+, **C-right**) human neurons in Flp-treated $APP^{+/+}$ and Cre-treated $APP^{Swe/+}$ cultures 5 weeks after induction. Data are from APP knockin clone H1.2. **(D)** Assessment of *APP* mRNA in $APP^{+/+}$ and $APP^{Swe/+}$ human neurons 10 days after Flp/Cre recombination with 2 sets of primers. **(E)** Representative immunoblots of APP protein in $APP^{+/+}$ and $APP^{Swe/+}$ human neuron lysates. For quantifications, see Fig. 1B. **(F-H)** Immunoblotting of Total and phosphor- Tau proteins in wild-type $APP^{+/+}$ and Swedish-mutant $APP^{Swe/+}$ human neurons (**F**, representative immunoblots of total and phosphorylated Tau in $APP^{+/+}$ and $APP^{Swe/+}$ human neuron lysates; **G** quantifications of total Tau protein; **H**; quantification of Tau phosphorylation by quantitative immunoblotting using the antibodies shown that react with Tau epitopes that are either part of, or related to, phospho-Tau; statistical significance for analyzed by 2-way ANOVA for the entire entire dataset, but direct comparisons do not exhibit a statistically significant difference). (**I**) Measurements of total Tau and pS396-Tau protein in lysates from matched human $APP^{+/+}$ and $APP^{Swe/+}$ neurons. **(J)** Representative images of sparsely transfected neurons used for quantifications of dendritic parameters. **(K)** Quantification of the total dendrite length, number of primary processes and number of branches of $APP^{+/+}$ and $APP^{Swe/+}$ human neurons in clone H1.1, H1.3 and 5d1.1. These data show for the clones listed measurements of the same parameters as demonstrated for clone H1.2 in Fig. 2A-B. **(L)** Quantification of cell body size of $APP^{+/+}$ and $APP^{Swe/+}$ human neurons in all clones. **(M)** Representative images of $APP^{+/+}$ and $APP^{Swe/+}$ human neurons derived from the conditionally $APP^{Swe/+}$-mutant iPS cell clone 5d1.1 immunostained for Rab5 (green) and MAP2 (red). Insets display magnified views of indicated fields. For quantifications, see Fig. **2D**. All data from human neurons analyzed 5 weeks after neuronal induction with Ngn2 unless otherwise noted. All numerical data are means ± SEM (numbers in bars are number of experiments (**D, G-I)** or number of cells or images/number of experiments analyzed (all other bar graphs)); their statistical significance was assessed by 2-way ANOVA (**H**) or Student's t-test (all other bar graphs) comparing matching $APP^{+/+}$ and $APP^{Swe/+}$ human neurons (*, $p<0.05$; **, $p<0.01$). Non-significant comparisons are not indicated.

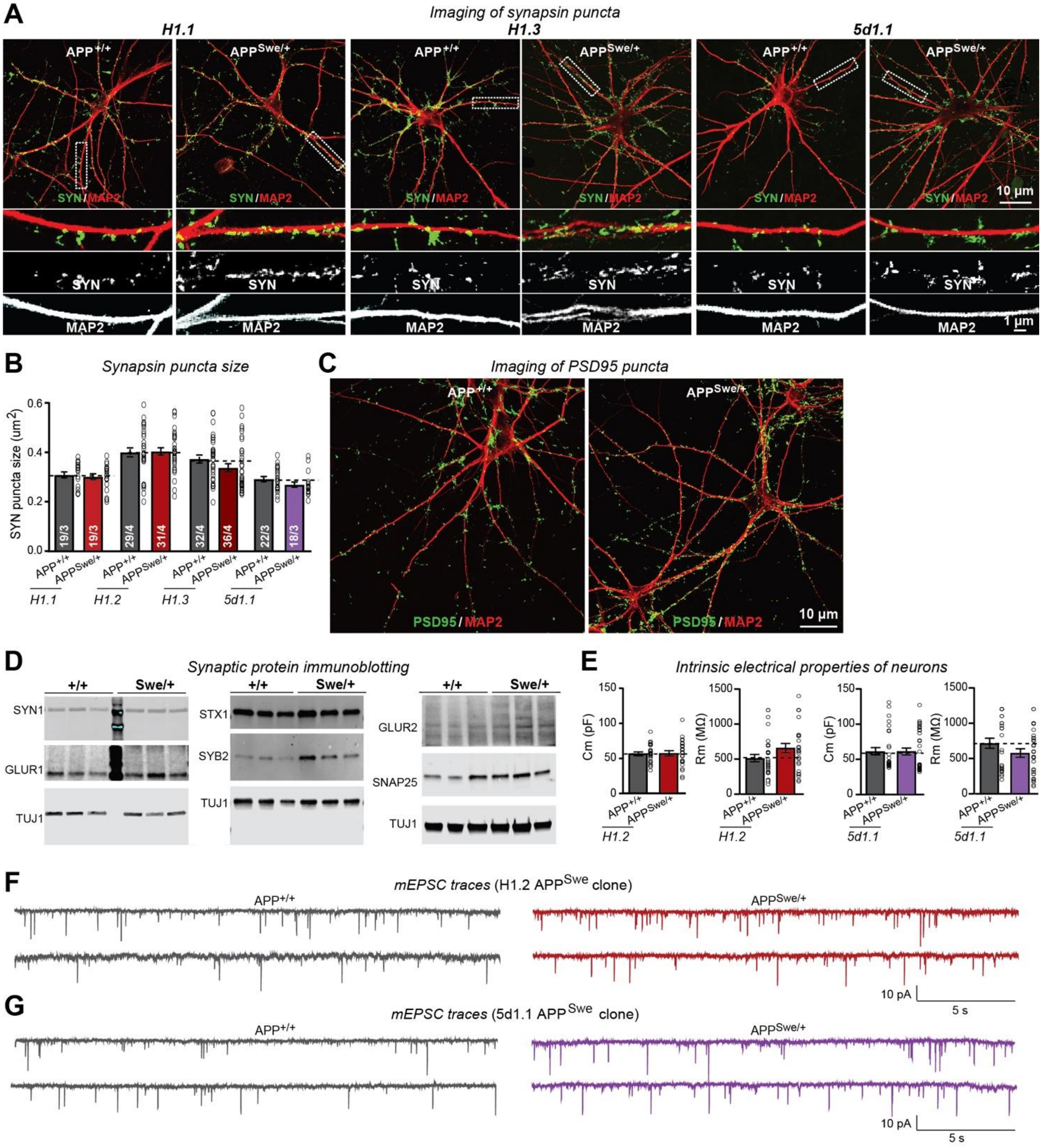

Fig. S4. Additional data on synapse formation and intrinsic electrical properties of APP$^{+/+}$ and APP$^{Swe/+}$ human neurons. **(A)** Representative images of 5-week-old APP$^{+/+}$ and APP$^{Swe/+}$ human neurons immunostained for Synapsin (SYN, green) and MAP2 (red). Lower panels are magnified views of the indicated fields showing both merged images and single-channel views. **(B)** Assessment of the Synapsin-positive puncta size in APP$^{+/+}$ and APP$^{Swe/+}$ human neurons from all 4 clones. For quantification of the synapse density, see Fig. 3F. **(C)** Representative images of APP$^{+/+}$ and APP$^{Swe/+}$ human neurons immunostained for PSD95 (green) and MAP2 (red). For quantification of the synapse density, see Fig. 3G. **(D)** Representative immunoblots of synaptic proteins in lysates of APP$^{+/+}$ and APP$^{Swe/+}$ human neurons. For quantifications, see Fig. 3H. **(E)** Membrane capacitance and input resistance of APP$^{+/+}$ and APP$^{Swe/+}$

human neurons derived from H1.2 and 5d1.1. (**F** & **G**) Additional representative mEPSC traces complementing those shown in Fig. 3A & D. All data from human neurons analyzed 5 weeks after neuronal induction with Ngn2; the number of cells or images/independent experiments analyzed are indicated in the bars of summary graphs. All numerical data are means ± SEM; their statistical significance was assessed by Student's t-test comparing matching $APP^{+/+}$ and $APP^{Swe/+}$ neurons. Non-significant comparisons are not indicated.

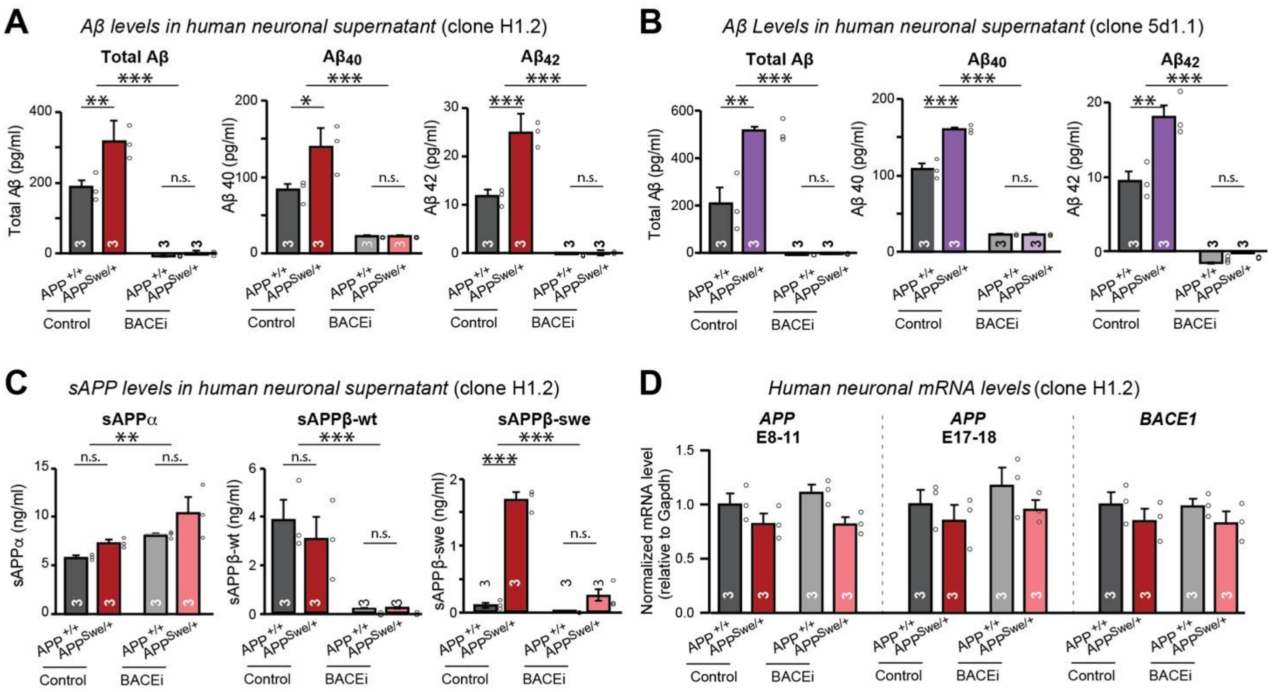

Fig. S5. Additional data demonstrating the effect of the BACE1 inhibitor LY2886721 (BACEi) on Aβ/sAPP secretion and on the relative concentration of *APP* mRNAs in human $APP^{+/+}$ and $APP^{Swe/+}$ neurons. (A & B) Inhibition of BACE1 by LY2886721 at 2 µM on human $APP^{+/+}$ and $APP^{Swe/+}$ neurons validated by Aβ peptides ELISA. Supernatants were harvested 17 days after neuronal induction in neurons derived from conditionally mutant ES cells (clone H1.2, **A**) and from conditionally mutant iPS cells (clone 5d1.1, **B**). **(C)** sAPP peptides secreted by $APP^{+/+}$ and $APP^{Swe/+}$ neurons with or without the BACE inhibitor treatments measured by ELISA in neurons derived from conditionally mutant ES cells (clone H1.2). **(D)** *APP* and *BACE1* mRNAs in $APP^{+/+}$ and $APP^{Swe/+}$ neurons with or without the BACE inhibitor treatments as measured by quantitative RT-PCR in human neurons 10 days after the recombination. The number of independent experiments is indicated in the bars. All data are means ± SEM; statistical significance was assessed by 2-way ANOVA with post-hoc corrections comparing matching $APP^{+/+}$ and $APP^{Swe/+}$ neurons (*, $p<0.05$; **, $p<0.01$; ***, $p<0.001$). Non-significant comparisons are not indicated.

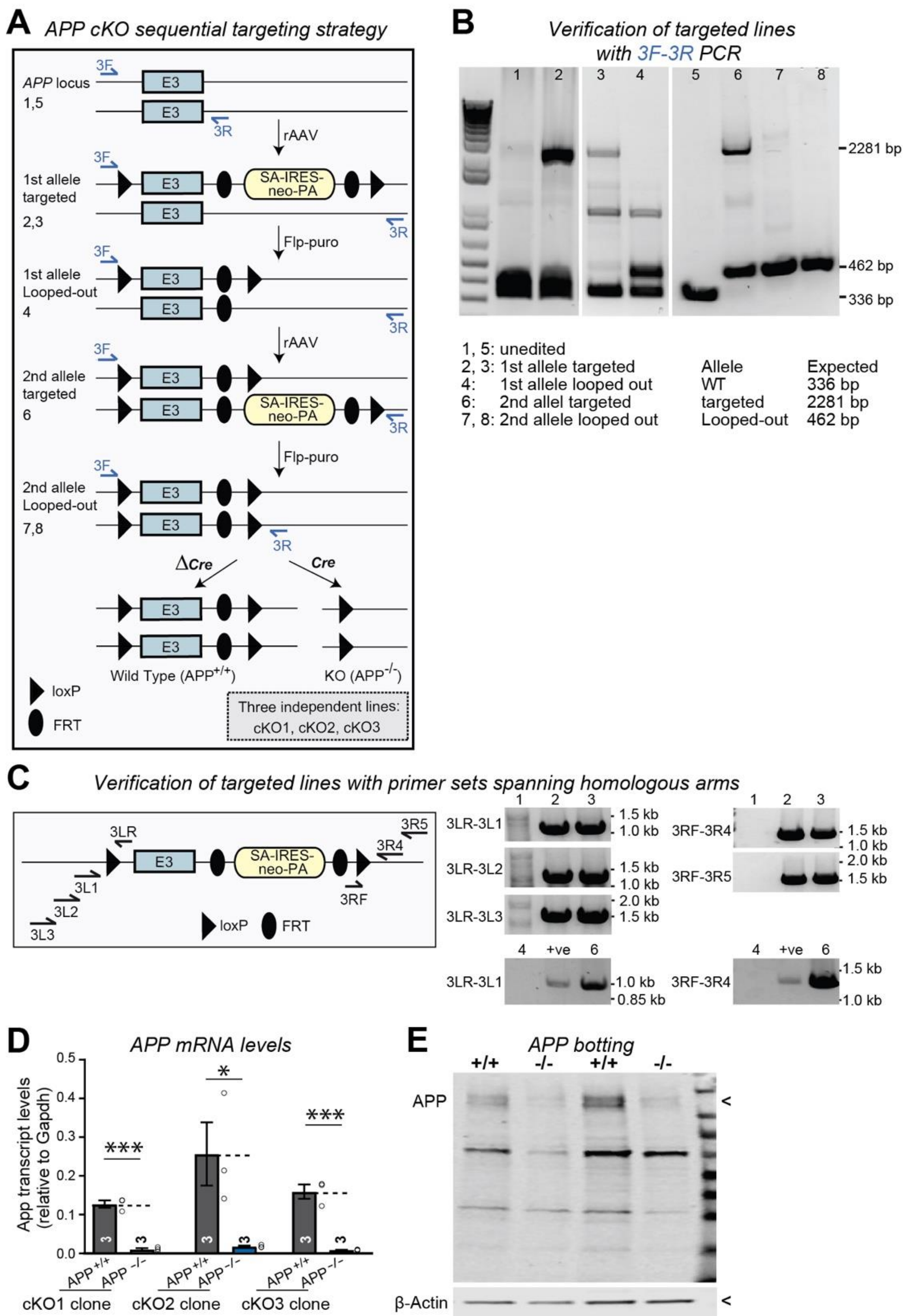

Fig. S6. Strategy for, and verification of, homozygous conditional KO mutations of *APP* in multiple engineered ES cell clones. (A) Targeting strategy. The two *APP* alleles were sequentially targeted via AAV-mediated homologous recombination as indicated. Upon looping out the IRES-neo cassettes, exon 3 of both alleles of the *APP* gene was floxed by loxP sites. Cre recombinase removes both *APP* exon 3 sequences and creates APP knockout ($APP^{-/-}$), while ΔCre retains wild-type *APP* alleles ($APP^{+/+}$). **(B)** PCR verification of targeted ES clones with the primer set 3F-3R indicated in a. Sample 1-8 were ES cell genomic DNAs harvested at different targeting steps as indicated. The PCR products from WT untargeted allele, targeted allele and looped-out alleles are 336 bp, 2281 bp, and 462 bp,

respectively. **(C)** PCR verification of targeted clones conducted with one primer on the loxP site (3LR and 3RF) and the other primer "external" to the homologous arms (Primer 3L1, 3L2, 3L3 for the left homologous arm, Primer 3R4, 3R5 for the right homologous arm). PCRs detect the correct recombination at both 5' and 3' sides beyond the targeting vector. Sample 1-6 were ES genomic DNAs harvested at different targeting steps as indicated in B. **(D)** Quantitative RT-PCR measuring *APP* mRNAin ΔCre-treated $APP^{+/+}$ and Cre-treated $APP^{-/-}$ human neurons 10 days after Cre recombination, as examined in a single representative experiment. **(E)** Representative immunoblots of APP in 5-week-old $APP^{+/+}$ and $APP^{-/-}$ human neuron lysates. The number of independent experiments is indicated in the bars in D. All data are means ± SEM; statistical significance was assessed by Student's t-test comparing matching $APP^{+/+}$ and $APP^{-/-}$ neurons (*, $p<0.05$; **, $p<0.01$; ***, $p<0.001$).

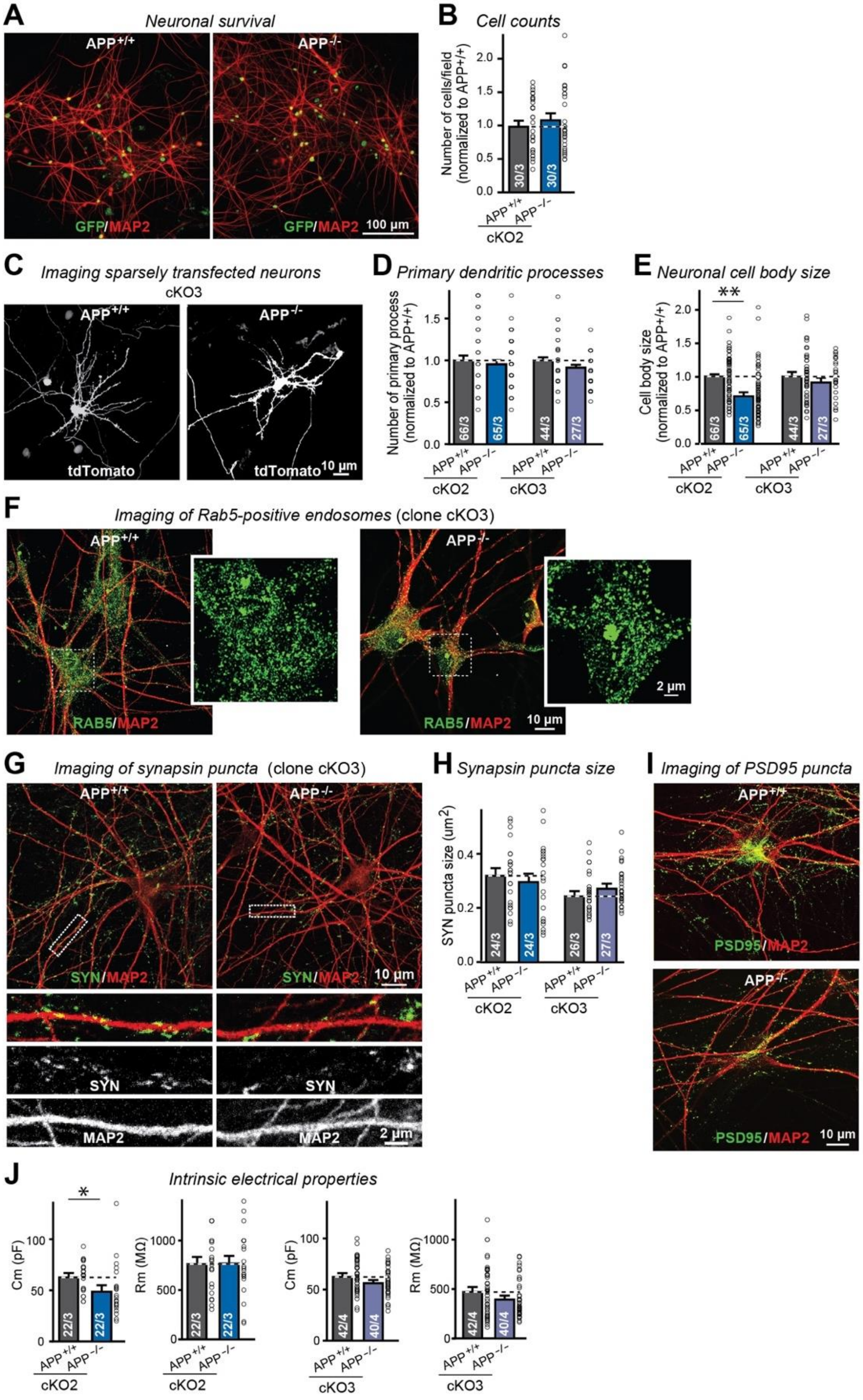

Fig. S7. Additional experiments characterizing human APP$^{-/-}$ neurons compared to their genetically precisely matched APP$^{+/+}$ controls that were derived from the same conditionally mutant parental ES cells line, including

dendrite development, synapse puncta sizes, and intrinsic electrical properties. **(A & B)** Representative images (**A**) and quantification of numbers per field (**B**) of MAP2+ human neurons in ΔCre -treated $APP^{+/+}$ and Cre-treated $APP^{-/-}$ cultures 5 weeks after induction. **(C)** Representative images of sparsely transfected ΔCre-treated $APP^{+/+}$ and Cre-treated $APP^{-/-}$ neurons used for quantification of dendritic parameters in Fig. 5E. **(D & E)** Quantification of the number of primary processes (**D**) and cell body size (**E**) of $APP^{+/+}$ and $APP^{-/-}$ human neurons. **(F)** Representative images of $APP^{+/+}$ and $APP^{-/-}$ human neurons derived from clone cKO3 immunostained with Rab5 (green) and MAP2 (red). Inserts in each image displayed magnified view of indicated fields. For quantifications, see Fig. 5G. **(G)** Representative images of $APP^{+/+}$ and $APP^{-/-}$ human neurons from cKO3 clone immunostained with SYN (green) and MAP2 (red). Lower panels are magnified views of indicated fields. For quantifications of the puncta density, see Fig. 6B. **(H)** Quantification of SYN puncta size in $APP^{+/+}$ and $APP^{-/-}$ human neurons derived from both clones. **(I)** Representative images of $APP^{+/+}$ and $APP^{-/-}$ human neurons immunostained with PSD95 (green) and MAP2 (red). For quantifications of the puncta density, see Fig. 6C. **(J)** Membrane capacitance and input resistance of $APP^{+/+}$ and $APP^{-/-}$ human neurons derived from clone cKO2 and cKO3. All data are from human neurons analyzed 5 weeks after neuronal induction with Ngn2 unless noted otherwise. All summary graphs means ± SEM (numbers in bars are number of cells or images/number of experiments analyzed; statistical significance was assessed by Student's t-test comparing matching $APP^{+/+}$ and $APP^{-/-}$ neurons (*, $p<0.05$; **, $p<0.01$). Non-significant comparisons are not indicated.

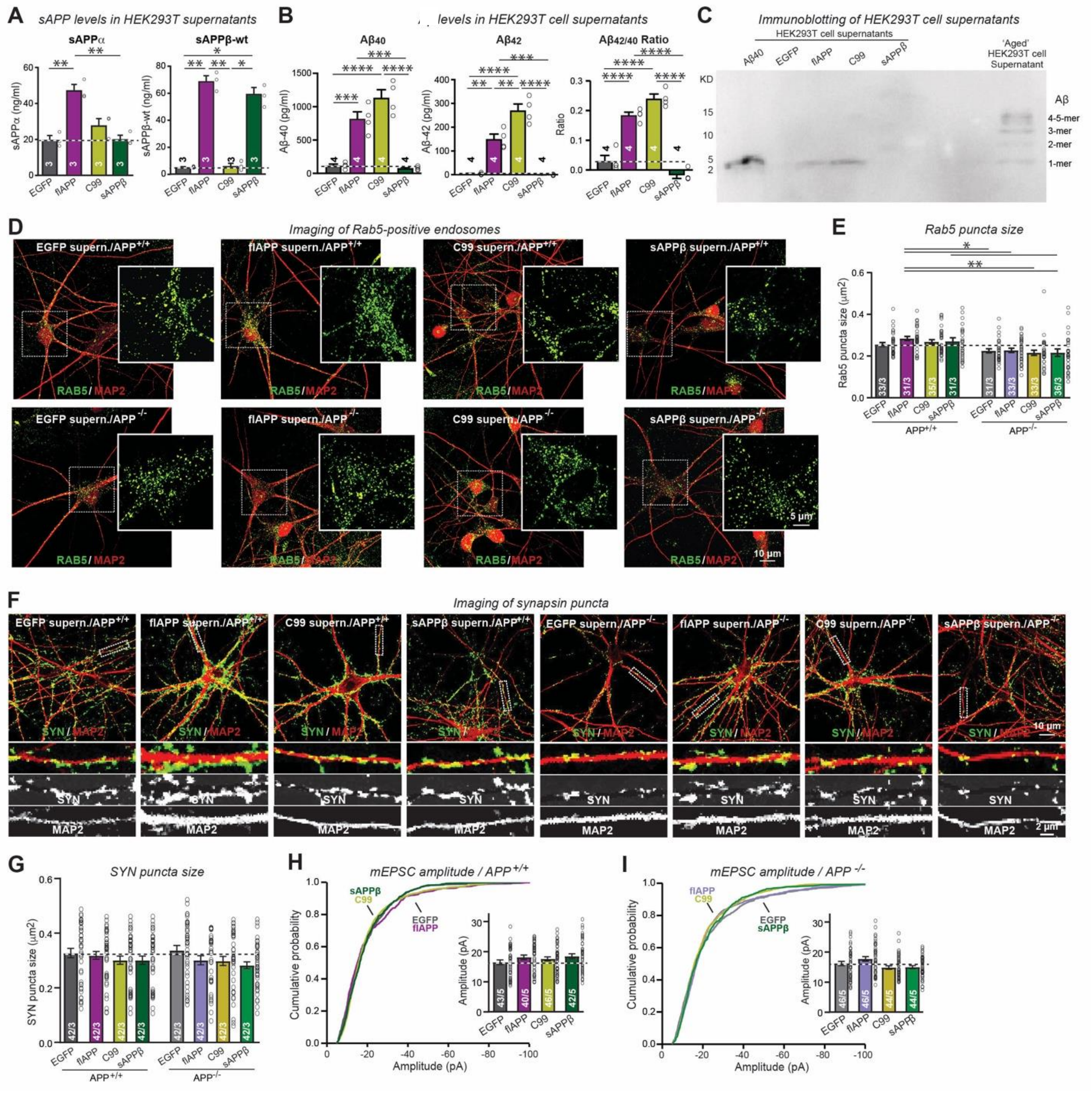

Fig. S8. Assessments of the secretion and aggregation of Aβ and sAPPβ that were obtained in the supernatants of transfected HEK293T cells, and measurements of their effects on the size of Rab5-positive endosomes, size of synaptic puncta, and mEPSC amplitudes in isogenic APP$^{+/+}$ and APP$^{-/-}$human neurons. **(A)** Measurements of sAPPα and sAPPβ in the supernatants of HEK293T cells transfected with the indicated APP expression plasmids. **(B)** Measurements of Aβ40, Aβ42, and the Aβ42/40 ratio in the supernatants of HEK293T cells transfected with the indicated APP expression plasmids. (**C**) Immunoblotting analysis of the multimerization state of Aβ in the fresh supernatant of transfected HEK293T cells expressing indicated APP plasmids. **(D & E)** Analysis of the Rab5-positive endosomes in human APP$^{+/+}$ and APP$^{-/-}$ neurons treated with supernatant of transfected HEK293T cells expressing indicated APP plasmids. (**D**, representative images of HEK293-cell supernatant-treated APP$^{+/+}$ and APP$^{-/-}$ human neurons derived from the cKO2 ES cell line; neurons were immunolabeled for Rab5 (green) and MAP2 (red); insets display magnified views of the indicated fields; **E**, quantification of the size of Rab5-positive endosomes). **(F & G)**

Quantification of the Synapsin-positive puncta sizes in human $APP^{+/+}$ and $APP^{-/-}$ neurons treated with the indicated HEK293T supernatants (**F**, representative images of $APP^{+/+}$ and $APP^{-/-}$ human neurons immunolabeled for Synapsin (SYN, green) and MAP2 (red); lower panels are magnified views of the indicated fields; **G**, quantification of the SYN puncta sizes). **(H & I)** Summary plots and cumulative probability graphs of the mEPSC amplitudes in $APP^{+/+}$ (**H**) and $APP^{-/-}$ (**I**) human neurons treated with the indicated HEK293T-cell supernatants containing elevated Aβ (flAPP, C99) or sAPPβ (flAPP, sAPPβ). All data are from human neurons analyzed 5 weeks after neuronal induction with Ngn2 unless noted otherwise. All summary graphs means ± SEM (numbers in bars are number of experiments (**A**) or number of cells or images/number of experiments analyzed (all other bar graphs)); statistical significance was assessed by one-way ANOVA (A) or 2-way ANOVA (all other bar graphs) with post-hoc corrections, or by KS test (insets in **H, I**) comparing matching $APP^{+/+}$ and $APP^{-/-}$ neurons (*, $p<0.05$; **, $p<0.01$). Non-significant comparisons are not indicated.

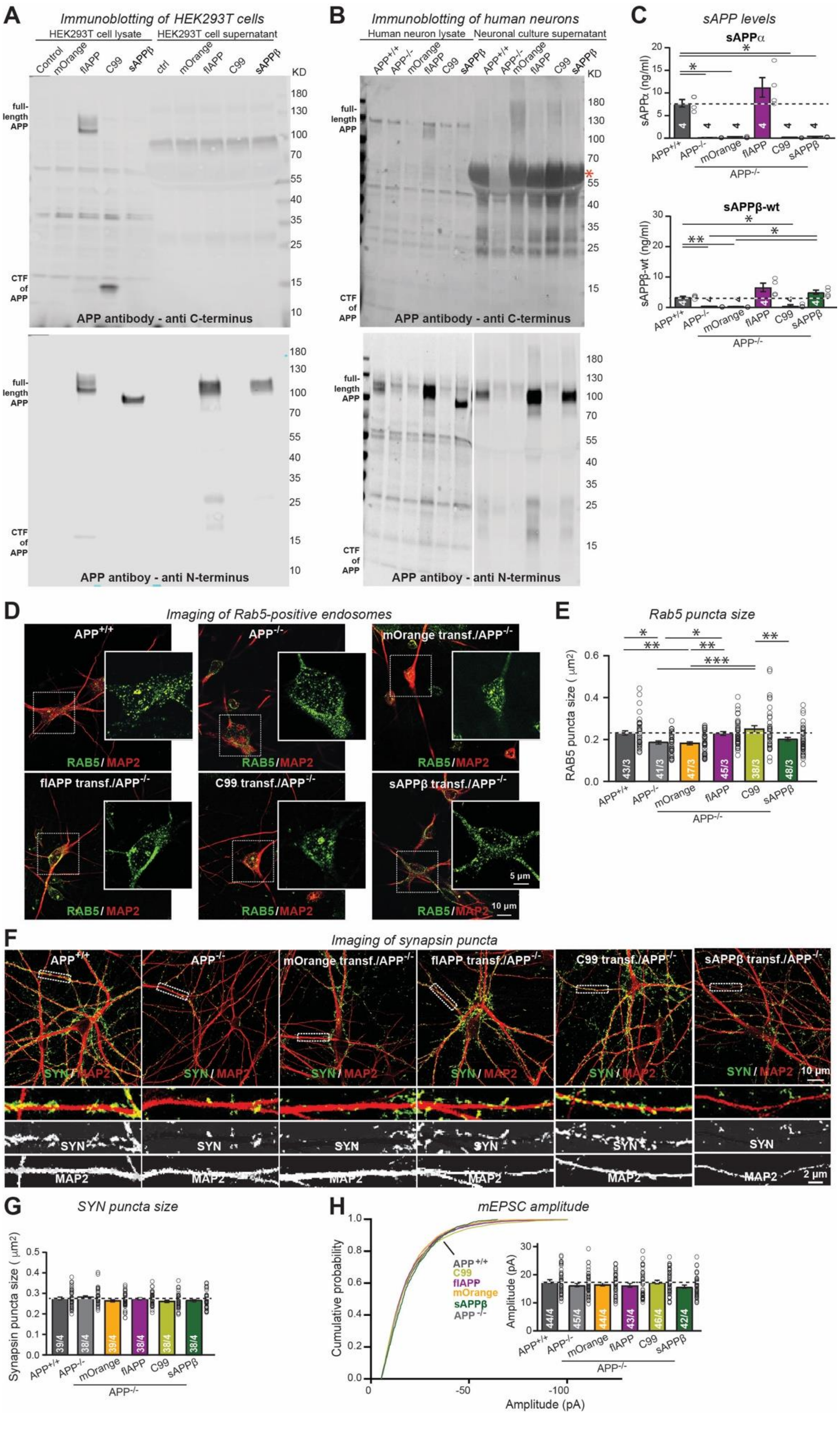

A
Immunoblotting of HEK293T cells
HEK293T cell lysate
HEK293T cell supernatant
Control
mOrange
flAPP
C99
sAPPβ
ctrl
KD
full-length APP
CTF of APP
APP antibody - anti C-terminus
APP antiboy - anti N-terminus
B
Immunoblotting of human neurons
Human neuron lysate
Neuronal culture supernatant
APP+/+
APP-/-
APP antibody - anti C-terminus
APP antiboy - anti N-terminus
C
sAPP levels
sAPPα
sAPPα (ng/ml)
sAPPβ-wt
sAPPβ-wt (ng/ml)
APP-/-
D
Imaging of Rab5-positive endosomes
APP+/+
APP-/-
mOrange transf./APP-/-
flAPP transf./APP-/-
C99 transf./APP-/-
sAPPβ transf./APP-/-
RAB5 / MAP2
5 µm
10 µm
E
Rab5 puncta size
RAB5 puncta size (µm2)
43/3
41/3
47/3
45/3
38/3
48/3
F
Imaging of synapsin puncta
SYN / MAP2
SYN
MAP2
10 µm
2 µm
G
SYN puncta size
Synapsin puncta size (µm2)
39/4
38/4
39/4
38/4
38/4
38/4
H
mEPSC amplitude
Cumulative probability
Amplitude (pA)
APP+/+
C99
flAPP
mOrange
sAPPβ
APP-/-
44/4
45/4
44/4
43/4
46/4
42/4

Fig. S9. Validation of the lentiviral expression of full-length APP (flAPP) or of the C99 and sAPPβ fragments of APP in HEK293T cells and human neurons, and measurements of the effects of the expression of full-length APP (flAPP) or of the C99 and sAPPβ fragments of APP on the size of Rab5-positive endosomes, size of synaptic puncta, and mEPSC amplitudes in human APP$^{-/-}$ neurons compared to precisely matched APP$^{+/+}$ controls. (A) Validation of expression viruses. Cell lysates and supernatants of HEK293T cells infected with lentiviruses expressing the indicated APP fragments were analyzed by immunoblotting with antibodies to the C- (top) and N-terminus (bottom) of APP. Control indicates uninfected HEK293T cell lysate and supernatant. **(B)** Immunoblots with antibodies recognizing C-terminal (top) and N-terminal (bottom) of APP on cell-lysates and supernatants of lentiviruses infected APP$^{-/-}$ human neurons. Uninfected APP$^{+/+}$ and APP$^{-/-}$ human neuron lysates and supernatants were included as further controls. **(C)** ELISA analysis measuring the sAPPα and sAPPβ in supernatants of human neurons infected with APP fragment overexpressing lentiviruses. **(D & E)** Assessment of the size of Rab5-positive endosomes in APP$^{-/-}$ human neuron overexpressing indicated APP fragments (**D**, representative images of infected APP$^{-/-}$ human neurons stained with Rab5 (green) and MAP2 (red); insets in each image display magnified view of indicated fields; **E**, quantification of the size of Rab5-positive endosomes). **(F & G)** Assessment of Synapsin-positive puncta sizes in APP$^{-/-}$ human neuron overexpressing indicated APP fragments (**F**, representative images of infected APP$^{-/-}$ human neurons immunolabeled with SYN (green) and MAP2 (red); lower panels are magnified views of indicated fields; **G**, quantification of the SYN puncta size). **(H)** Summary plots and graphs of the mEPSCs amplitude in infected APP$^{-/-}$ human neurons. All data are from human neurons analyzed 5 weeks after neuronal induction with Ngn2 unless noted otherwise. Uninfected APP$^{+/+}$ and APP$^{-/-}$ neurons were used as further controls for all the comparisons. All summary graphs means ± SEM (numbers in bars are number of experiments (**C**) or number of cells or images/number of experiments analyzed (all other bar graphs)); statistical significance was assessed by one-way ANOVA with post-hoc corrections (all bar graphs) or KS test (inset in **H**) (*, $p<0.05$; **, $p<0.01$; ***, $p<0.001$). Non-significant comparisons are not indicated.

Table S1. Individual subject-level data for statistics with n<20. (See supporting files)

Table S2. Key resources table.

**Reagent**	**Source**	**Identifier**
**Antibodies (names list targets)**		
APP	Südhof lab	U955
APP	Sigma	MAB348
β-Amyloid	Cell Signaling	D54D2
Synapsin-1	Südhof lab	E028
Synapsin-1	YenZym	YZ6078
MAP2	Abcam	AB5392; RRID:AB_2138153
MAP2	Encor	CPCA-MAP2
PSD95	ThermoFisher	MA1-046; RRID:AB_2092361
Homer-1b/c	Yenzyme	YZ6085
Synaptophysin-1	Jahn Monoclonals	C7.2
Synaptotagmin-1	Hybridoma Bank	mAB 48 (asv 48)
vGLUT1	Neuromab	75-006
GFP	Abcam	ab13970; RRID:AB_300798
RAB5	Cell Signaling Techn.	3547
TUJ1	BioLegend	MMS-435P
GluA1	Neuromab	75-327
GluA2	Neuromab	75-002
SNAP25	Südhof lab	P913
Synaptobrevin-2	SYSY	104 211
Syntaxin	Südhof lab	438B
AT8	ThermoFisher	MN1020
AT270	ThermoFisher	MN1050
PHF1	Dr. Peter Davies lab	N/A
MC1	Dr. Peter Davies lab	N/A
TAU	Simga	MAB361
β-ACTIN	Sigma	A1978
**Chemicals, Peptides, and Recombinant Proteins**		
QX-134	Tocris	1014
Picrotoxin	Tocris	1128
Tetrodotoxin	Amer. Radiol. Chemicals	ARCD 0640
mTeSR1 medium	Stem Cell Technologies	85850
StemFlex™ Medium	Thermo Fisher	A3349401
DMEM/F-12	Thermo Fisher	11320033
Neurobasal™ Medium	Thermo Fisher	21103049
DMEM	Thermo Fisher	11995
N-2 Supplement	Thermo Fisher	17502048
B-27™ Supplement	Thermo Fisher	17504044
HyClone FBS	Thermo Fisher	SH30071.03
MEM NEAA Solution	Thermo Fisher	11140050
GlutaMAX™	Thermo Fisher	35050061
Sodium Pyruvate	Thermo Fisher	11360070
Accutase	Innovative Cell Tech.	AT-104
Trypsin-EDTA (0.25%)	Thermo Fisher	25200056
Papain, Suspension	Worthington Biochem	LS003127
Matrigel Membrane Matrix	Corning	CB-40234A
Thiazovivin	BioVision	1681
Doxycycline hyclate	Sigma	D9891
Puromycin (endotoxin free)	Invivogen	ant-pr
Polyethylenimine (PEI)	Polysciences Inc.	23966

Benzonase	Sigma	70664
LY2886721	Cayman Chemical	21599
Tau (Total) Human	Thermo Fisher	KHB0041
Tau (Phospho) [pS396] Human	Thermo Fisher	KHB7031
ELISA human Aβ40	Thermo Fisher	KHB3481
ELISA human Aβ42	Thermo Fisher	KHB3544
ELISA human total Aβ	Takara Bio	27729
Human sAPPα Assay Kit	Takara Bio	27734
Human sAPPβ-w Assay Kit	Takara Bio	27732
Human sAPPβ-sw Assay Kit	Takara Bio	27733
Direct-zol RNA Kits	Zymo	R2051
DNase I Set	Zymo	E1010
One-Step qRT-PCR Master Mix	Affymetrix	78350
**Experimental Models: Cell Lines**		
HEK293T/17 cells	ATCC	CRL-11268; RRID: CVCL_0063
H1 ES cell line	WiCell Res. Inst., Inc.	WA01; RRID: CVCL_9771
Human iPS cell line 5d1	Dr. Tanabe I-PEASE, Inc.	N/A
**Experimental Models: Organisms/Strains**		
CD1 mice	Charles River	Cat. No. 22
**Oligonucleotides**		
TaqMan qPCR assay GAPDH	IDT	Hs.PT.58.40035104
TaqMan qPCR assay ACTIN-b	IDT	Hs.PT.39a.22214847
TaqMan qPCR assay APP E8-11	IDT	Hs.PT.56a.274278414
TaqMan qPCR assay APP E17-18	IDT	Primer 1: ggtgtggtggaggttgac<br>Primer 2: agttctgcatctgctcaaaga<br>Probe: agatgcagcagaacggctacgaaa
TaqMan qPCR assay BACE1	IDT	Hs.PT.58.5050046
TaqMan qPCR assay MAP2	IDT	Hs.PT.58.25625979
TaqMan qPCR assay TUJ1	IDT	Hs.PT.58.20385221
TaqMan qPCR assay PSD95	IDT	Hs.PT.58.20575145
TaqMan qPCR assay SYT1	IDT	Hs.PT.58.19615550
TaqMan qPCR assay SYN1	IDT	Hs.PT.58.1323901
TaqMan qPCR assay CASK	IDT	Hs.PT.58.26646974
TaqMan qPCR assay APOE	IDT	Hs.PT.58.28344970
TaqMan qPCR assay VGLUT1	IDT	Hs.PT.58.22392732
TaqMan qPCR assay VGAT	IDT	Hs.PT.58.4211177
TaqMan qPCR assay GPHRN	IDT	Hs.PT.58.234679
TaqMan qPCR assay NLGN1	IDT	Hs.PT.58.20755367
TaqMan qPCR assay NLGN2	IDT	Hs.PT.58.3800688
TaqMan qPCR assay GAD1	IDT	Hs.PT.58.3646327
TaqMan qPCR assay GAD2	IDT	Hs.PT.58.26176171
TaqMan qPCR assay OCT4	IDT	Hs.PT.58.14648152.g
TaqMan qPCR assay SOX2	IDT	Hs.PT.58.45375945.g
TaqMan qPCR assay NANOG	IDT	Hs.PT.58.21480849
**Recombinant DNA**		
FUW-TetO-Ngn2- P2A-puro	Zhang et al., 201344	N/A
FUW-rtTA	Zhang et al., 201344	N/A
FUW-GFP::Cre (lenti vect.)	Yi et al., 201659 [61]	N/A
FUW-GFP::Flp (lenti vect.)	This paper	N/A
FUW-GFP::ΔCre (lenti vect.)	Yi et al., 201659	N/A
pAAV	Lisowski et al., 201460	N/A
pAd5	Lisowski et al., 201460	N/A
pRSV-rev	Addgene	12253

pMDLg/ pRRE	Addgene	12251
pVSV-G	Addgene	138479
FCW-EGFP	Marro et al., 210961	N/A
FCW-tdTomato	Addgene	83029
pCAX-IRES-EGFP	This paper	N/A
pCAX-APP-695	Addgene	30137
pCAX-APP-C99	Addgene	30146
pCAX-APP-sAPPβ-WT	This paper	N/A
FCW-Orange (lenti vect.)	This paper	N/A
FCW-full_length-APP (lenti vect.)	This paper	N/A
FCW-APP-C99 (lenti vect.)	This paper	N/A
FCW-APP-sAPPβ-WT (lenti vect.)	This paper	N/A
**Software and Algorithms**		
GraphPad Prism 8	GraphPad	https://www.graphpad.com
Adobe Illustrator	Adobe	https://www.adobe.com
pCLAMP 10.7	Molecular Devices	https://www.moleculardevices.com
Image Studio 5.2.5	LI-COR Biosci.	https://www.licor.com/bio
FIJI/ImageJ	NIH	https://imagej.nih.gov/ij
Metamorph	Molecular Devices	https://www.moleculardevices.com
NIS-Elements Confocal	Nikon	https://www.nikoninstruments.com
ZEN	Zeiss	https://www.zeiss.com/microscopy
